# Genome-Wide Pharmacogenomic Study on Methadone Maintenance Treatment Identifies SNP rs17180299 and Multiple Haplotypes on *CYP2B6*, *SPON1*, and *GSG1L* Associated with Plasma Concentrations of Methadone *R-* and *S-*enantiomers in Heroin-Dependent Patients

**DOI:** 10.1371/journal.pgen.1005910

**Published:** 2016-03-24

**Authors:** Hsin-Chou Yang, Shih-Kai Chu, Chieh-Liang Huang, Hsiang-Wei Kuo, Sheng-Chang Wang, Sheng-Wen Liu, Ing-Kang Ho, Yu-Li Liu

**Affiliations:** 1 Institute of Statistical Science, Academia Sinica, Taipei, Taiwan; 2 Bioinformatics Program, Taiwan International Graduate Program, Institute of Information Science, Academia Sinica, Taipei, Taiwan; 3 Institute of Public Health, National Yang Ming University, Taipei, Taiwan; 4 Department of Statistics, National Cheng-Kung University, Tainan, Taiwan; 5 School of Public Health, National Defense Medical Center, Taipei, Taiwan; 6 Institute of Biomedical Informatics, National Yang-Ming University, Taipei, Taiwan; 7 Center for Drug Abuse and Addiction, China Medical University Hospital, Taichung, Taiwan; 8 School of Medicine, China Medical University, Taichung, Taiwan; 9 Center for Neuropsychiatric Research, National Health Research Institutes, Miaoli County, Taiwan; 10 Graduate Institute of Clinical Medical Science, China Medical University, Taichung, Taiwan; Stanford University School of Medicine, UNITED STATES

## Abstract

Methadone maintenance treatment (MMT) is commonly used for controlling opioid dependence, preventing withdrawal symptoms, and improving the quality of life of heroin-dependent patients. A steady-state plasma concentration of methadone enantiomers, a measure of methadone metabolism, is an index of treatment response and efficacy of MMT. Although the methadone metabolism pathway has been partially revealed, no genome-wide pharmacogenomic study has been performed to identify genetic determinants and characterize genetic mechanisms for the plasma concentrations of methadone *R*- and *S*-enantiomers. This study was the first genome-wide pharmacogenomic study to identify genes associated with the plasma concentrations of methadone *R*- and *S*-enantiomers and their respective metabolites in a methadone maintenance cohort. After data quality control was ensured, a dataset of 344 heroin-dependent patients in the Han Chinese population of Taiwan who underwent MMT was analyzed. Genome-wide single-locus and haplotype-based association tests were performed to analyze four quantitative traits: the plasma concentrations of methadone *R*- and *S*-enantiomers and their respective metabolites. A significant single nucleotide polymorphism (SNP), rs17180299 (raw *p* = 2.24 × 10^−8^), was identified, accounting for 9.541% of the variation in the plasma concentration of the methadone *R*-enantiomer. In addition, 17 haplotypes were identified on *SPON1*, *GSG1L*, and *CYP450* genes associated with the plasma concentration of methadone *S*-enantiomer. These haplotypes accounted for approximately one-fourth of the variation of the overall *S*-methadone plasma concentration. The association between the *S*-methadone plasma concentration and *CYP2B6*, *SPON1*, and *GSG1L* were replicated in another independent study. A gene expression experiment revealed that *CYP2B6*, *SPON1*, and *GSG1L* can be activated concomitantly through a constitutive androstane receptor (CAR) activation pathway. In conclusion, this study revealed new genes associated with the plasma concentration of methadone, providing insight into the genetic foundation of methadone metabolism. The results can be applied to predict treatment responses and methadone-related deaths for individualized MMTs.

## Author Summary

Methadone maintenance treatment (MMT), among the most effective therapies for heroin-dependent patients, reduces craving and withdrawal symptoms, increases treatment compliance, and improves the quality of life of patients. The plasma concentration of methadone is a primary index for quantifying and determining therapy responses to MMT. This study was the first whole-genome pharmacogenomic study on MMT to locate genomic regions associated with the plasma concentration of methadone. The analysis identified a single nucleotide polymorphism (SNP) marker (rs17180299) and 17 haplotypes on the *SPON1*, *GSG1L*, and *CYP450* genes, including *CYP2B6* significantly associated with the plasma concentrations of methadone enantiomers. The identified genetic variations accounted for approximately 10% and 25% of the variations in plasma concentrations of methadone *R*- and *S*-enantiomers, respectively. The identified genetic variations have afforded insight into the genetic mechanism of the metabolism of MMT, and have potential to pave the way towards individualized MMTs for heroin-dependent patients.

## Introduction

Heroin dependence is a severe psychiatric disorder characterized by a heroin craving behavior and the inability to stop using heroin. The World Health Organization reported that there were more than 9 million heroin users in 2014, and the prevalence of heroin dependence is increasing globally. Heroin abuse can incur medical complications and generate social problems; these problems have substantially increased the burden on social security and health insurance systems [1, 2].

Replacement or maintenance therapy with an opioid analog is among the most commonly used treatments for heroin dependence, and reduces craving and withdrawal symptoms, increases treatment compliance, and improves the quality of life of patients [3]. Methadone, a synthetic opioid, is a commonly used medication for treating heroin dependence [4]. Methadone maintenance treatment (MMT) reportedly retains patients and decreases heroin use more effectively than no treatment [4–6].

The chemical structure of methadone has a chiral center that produces the racemic enantiomers of *R*- and *S*-form [7]. Methadone *R*- and *S*-enantiomers have different metabolic preferences for the liver cytochrome P-450 (CYP450) isozyme [8]: the CYP2C19 isozyme metabolizes methadone *R*-enantiomer [8, 9] and CYP2B6 isozyme metabolizes methadone *S*-enantiomer [10, 11]. Several pharmacogenetic studies have found that the genetic variants located in the *CYP2B6* region were associated with methadone [12] and could be observed to predict methadone-related deaths [13, 14]. Through an interaction between *CYP3A4* and *CYP2C19*, a genetic matrix exhibited a titration prediction effect for the required methadone dose [15].

The steady-state plasma concentration of methadone is an index for quantifying methadone metabolism and can serve as a surrogate marker for the treatment responses and efficacy of MMT. Previous pharmacogenetic studies have reported the association between *CYP2B6* and the plasma concentration of methadone *S*-enantiomer [16–18]. We previously established a system used to measure methadone enantiomers and their metabolism product, metabolite 2-ethylidene-1,5-dimethyl-3,3-diphenyl pyrrolidine (EDDP) [9]. Pharmacogenetic studies on the *CYP2B6* gene in a methadone maintenance cohort in Taiwan revealed the association between the plasma concentration of methadone *S*-enantiomer and single nucleotide polymorphisms (SNPs) located at the region of *CYP2B6* gene [16]. In addition, the pregnane X receptor, a steroid and xenobiotic sensing nuclear receptor, possibly interacts with genetic variants in *CYP2B6* to associate with the plasma concentration of methadone *S*-enantiomer [19]. In addition to genes in the *CYP450* gene family, *ABCB1* was reported to be associated with the plasma concentration of methadone [20]. However, the association remains unclear because the finding was not replicated in other studies [18, 21]. Dennis et al. provided a systematic review on the effect of *ABCB1* and *CYP2B6* on methadone metabolism [18].

Although the methadone metabolism pathway has been partially revealed [22], there is still no report of genome-wide association analyses that characterize the plasma concentrations of the methadone *R*- and *S*-enantiomers. Moreover, the metabolism regulatory genes associated with the plasma concentration of methadone remain unclear. Previous pharmacogenetic studies on the plasma concentration of methadone enantiomer have used a candidate gene approach and single locus association analyses [16, 17]. Because of the clinical importance of treatment responses to MMT and lack of results from genome-wide pharmacogenomic research, the aim of this study was to conduct the first genome-wide pharmacogenomic investigation of the plasma concentrations of methadone enantiomers and their metabolites. We recruited and genotyped a Han Chinese methadone maintenance cohort comprising 360 heroin-dependent patients in Taiwan by using the Axiom Genome-Wide CHB 1 Array. Genome-wide single-locus and multilocus methods were applied to identify the susceptibility loci and their contribution in the variability of the methadone plasma concentration. Furthermore, an independent methadone maintenance cohort comprising 78 heroin-dependent patients under the same inclusion and exclusion criteria was recruited to replicate the susceptibility loci identified in the genome-wide pharmacogenomic study (discovery stage). In addition to identifying novel genes associated with the plasma concentration of methadone, this pharmacogenomic study was the first to verify the results of previous biological metabolic tests and the methadone pathways.

## Results

### Quality control

**Fig 1:**
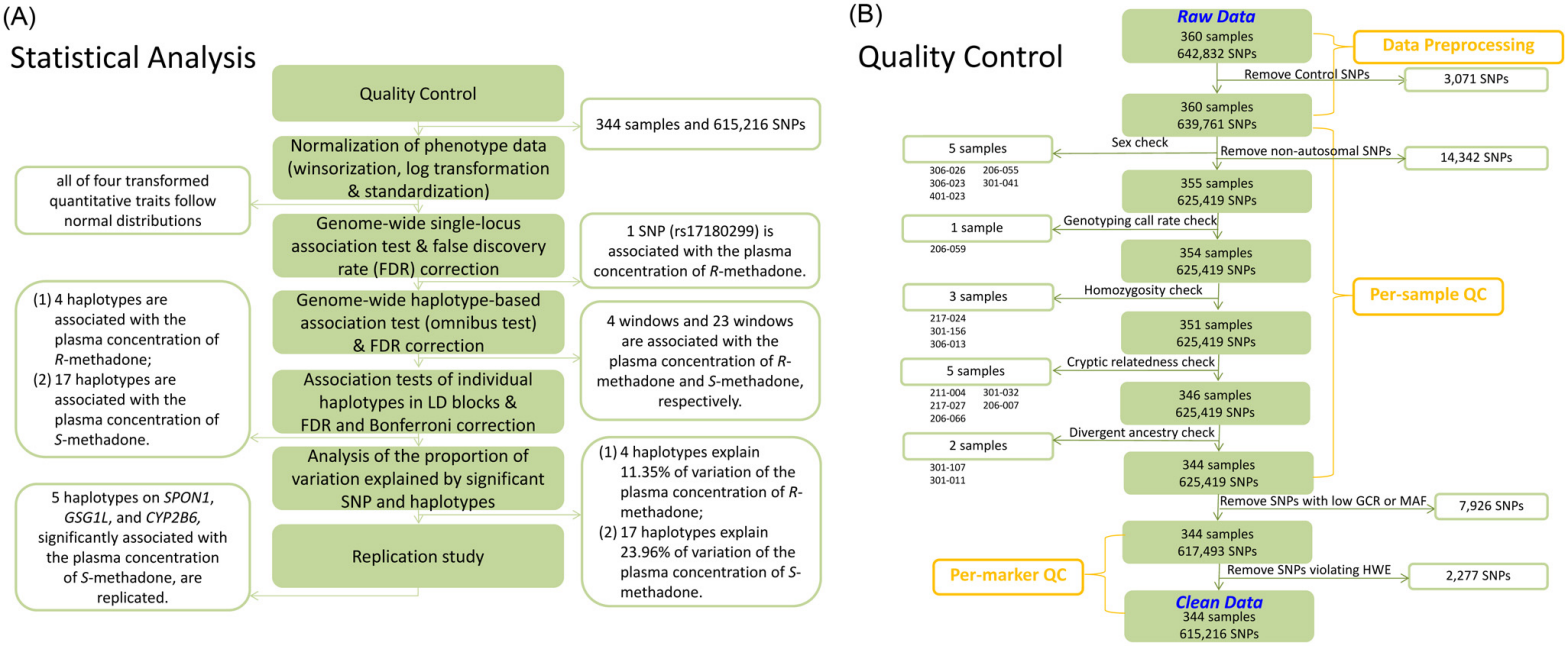
Analysis flow of this study. (**A**) Flow of all statistical analyses. (**B**) Flow of quality control.

Fig 1(A) shows flow charts and results of all statistical analyses, and Fig 1(B) depicts the results of quality control. In total, 360 MMT patients were genotyped with the Axiom Genome-Wide CHB 1 Array. Five patients (306–026, 306–023, 401–023, 206–055, and 301–041), who exhibited a homozygosity pattern of X chromosome(s) inconsistent with their self-reported gender, were removed (S1 Fig). One patient (206–059) was removed because of a low genotyping call rate (GCR) of <0.95. Three patients (217–024, 301–156, and 306–013) with an overly high genome-wide homozygosity rate were removed (S2 Fig). Two of the three patients (217–024, S3(A) Fig; 301–156, S3(B) Fig) with an overly high genome-wide homozygosity rate were found to have excessive runs of homozygosity on multiple chromosomes (S3(C) Fig). Five pairs of individuals exhibiting cryptic relatedness were identified; the identity-by-descent (IBD) estimates were 0.5891, 0.5052, 0.4431, 0.4571, and 0.4641. We removed five individuals with the lower GCR in each pair (211–004, 217–027, 206–066, 301–032, and 206–007). Finally, we removed two patients (301–107 and 301–011) who exhibited divergent ancestry; their first two principal components of allele frequency were outside the confidence bands of our population, the Han Chinese population in Taiwan (S4 Fig). In total, 16 individuals were removed from the subsequent association analyses. After the quality examination of SNP, 14,342 nonautosomal SNPs were removed, 7,926 SNPs with a GCR of <0.95 or a minor allele frequency (MAF) of <0.01 were removed, and 2,277 additional SNPs were removed because of a deviation from the Hardy-Weinberg equilibrium (HWE) (*p* < 8.1 × 10^−8^). In this study, 615,216 valid SNPs (approximately 98% of the autosomal SNPs on the Axiom Genome-Wide CHB 1 Array) were analyzed.

### Three covariates and four quantitative traits

We examined three covariates (age, sex, and body mass index [BMI]) and four quantitative traits (the plasma concentrations of the methadone *R*- and *S*-enantiomers and their metabolites). Based on the post-quality-control data, the distributions of the three covariates and four quantitative traits are summarized in Table 1. Among the male patients, the mean age and BMI were 39.3061 (standard deviation [SD] = 7.6587) and 23.9176 (SD = 3.4537), respectively. Among the female patients, the mean age and BMI were 33.0318 (SD = 5.4506) and 22.3980 (SD = 3.5792), respectively. The normality of the quantitative trait was a critical assumption required for the association analysis. The normality of the four quantitative traits was examined; Table 1 shows a summary of the results. Before any data transformation, the Kolmogorov–Smirnov goodness-of-fit tests for normality [23] revealed that the four quantitative traits significantly violated normality. After data transformation by winsorization, log transformation, and standardization, the normality assumption was achieved for all of the plasma concentrations of *R*-methadone (*p* = 0.6255), *S*-methadone (*p* = 0.0802), *R*-EDDP (*p* = 0.7903), and *S*-EDDP (*p* = 0.1876). Data distributions of the four quantitative traits before and after data transformation are displayed in S5(A) and S5(B) Fig, respectively.

**Table 1:** Summary statistics of covariates and the raw data and transformed data of quantitative traits by gender. The number of individuals (i.e., sample size), means ± standard deviations (SD) for covariates and quantitative traits are provided. The final column provides the *p* value of the Kolmogorov-Smirnov Good-of-Fit test for normality for the raw data and transformed data of the four quantitative traits.

	Male		Female		
Characteristics	Sample size	Mean ± SD	Sample size	Mean ± SD	Normality test (*p* value)
Age (years)	281	39.3061 ± 7.6587	63	33.0318 ± 5.4506	-
BMI (kg/m^2^)	278	23.9176 ± 3.4537	63	22.398 ± 3.5792	-
Raw plasma *R*-methadone/dose (ng/ml/mg)	281	3.871 ± 2.4659	63	3.755 ± 1.7789	1.41×10^−6^
Raw plasma *S*-methadone/dose (ng/ml/mg)	281	2.787 ± 1.5619	63	2.566 ± 1.4909	2.67×10^−3^
Raw plasma *R*-EDDP/dose (ng/ml/mg)	272	0.2975 ± 0.455	63	0.2964 ± 0.4587	< 2.2×10^−16^
Raw plasma *S*-EDDP/dose (ng/ml/mg)	277	0.3286 ± 0.5477	63	0.282 ± 0.1842	< 2.2×10^−16^
Transformed plasma *R*-methadone/dose (ng/ml/mg)	281	0.0030 ± 0.9946	63	-0.0136 ± 1.0316	0.6255
Transformed plasma *S*-methadone/dose (ng/ml/mg)	281	0.0173 ± 1.0227	63	-0.0774 ± 0.8953	0.0802
Transformed plasma *R*-EDDP/dose (ng/ml/mg)	272	0.0212 ± 0.9787	63	-0.0916 ± 1.0908	0.7903
Transformed plasma *S*-EDDP/dose (ng/ml/mg)	277	-0.0004 ± 1.0075	63	0.0019 ± 0.9741	0.1876

### Genome-wide singe-locus association analysis

**Fig 2:**
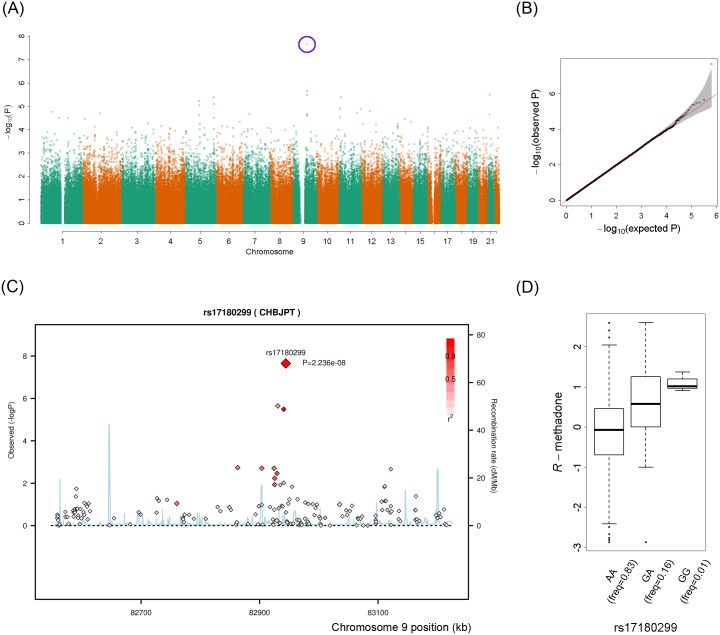
Genome-wide single-locus association test for plasma concentration of *R*-methadone. (**A**) Manhattan plot of genome-wide single-locus association test. The vertical axis indicates the raw *p* values of the association tests (in a scale of -log10). The horizontal axis is physical position of a SNP on different autosomes. Each point indicates the raw *p* value of a SNP. A significant SNP is circled in blue. (**B**) Quantile–Quantile plot of genome-wide single-locus association test. The vertical axis indicates the raw *p* values of the association tests (in a scale of -log10). The horizontal axis indicates the expected *p* values of the association tests (in a scale of -log10) under a null hypothesis, i.e., no genetic association. (**C**) Regional association plot of the significant SNP, rs17180299. The vertical axis indicates the raw *p* values of the association tests (in a scale of -log10). The horizontal axis is physical position of SNP (in unit of kb). (**D**) Distribution of plasma concentration of *R*-methadone for three genotypes of rs17180299.

As shown in the Manhattan plot (Fig 2(A)) and Quantile–Quantile plot (Fig 2(B)), the genome-wide single-locus association analysis identified only SNP rs17180299 to be significantly associated with the plasma concentration of *R*-methadone after a multiple-test correction of a false discovery rate (raw *p* = 2.24 × 10^−8^). Fig 2(C) depicts the regional association plot for the flanking genomic region of SNP rs17180299 on chromosome 9. Rs17180299 was an *A*/*G* polymorphism; the frequency of minor allele *G* was 0.09. A trend of the dose-response effect of rs17180299 on the plasma concentration of *R*-methadone was observed (Fig 2(D)). Patients who had more allele *G* tended to have a higher plasma concentration of *R*-methadone. A SNP cluster plot showed that individuals with different genotypes were clearly separated, implying that the genotype calls were reliable. However, this SNP was located in an intergenic region.

For the other three traits, no single SNP was significant after a multiple-test correction of the false discovery rate. The Manhattan plots of genome-wide SNPs (in a scale of -log_10_) for the plasma concentration of *S*-methadone (S6(A) Fig), the plasma concentration of *R*-EDDP (S6(B) Fig), and the plasma concentration of *S*-EDDP (S6(C) Fig) were created. The SNPs with the smallest *p* values in the genome-wide association analysis of the plasma concentration of *S*-methadone, *R*-EDDP, and *S*-EDDP were AX-16534452 (raw *p* = 4.83 × 10^−7^), rs1448332 (raw *p* = 8.18 × 10^−7^), and AX-13599782 (raw *p* = 2.08 × 10^−6^), respectively. The SNP AX-16534452 was located on *SPON1* on chromosome 11. The SNPs rs1448332 and AX-13599782 were located in an intergenic region on chromosome 3 and chromosome 21, respectively.

### Genome-wide haplotype-based association analysis

We performed sliding-window haplotype-based association analyses with a window size of 2, 3, 4, 5, and 10 individually. For each window size, we separately performed haplotype-based association analyses for the four quantitative traits, adjusting for age, sex, and BMI. Only the results on a basis of a window size of 5 were reported because of the following two reasons. First, the locations of significant haplotypes in the haplotype-based association analyses of different window sizes were quite consistent. Among the 25 most significant windows in each of the analyses of different window sizes, higher than 92.5% of the significant windows were overlapped. Second, in the study population, a window size of 5 provided a distribution of window width nearest to the distribution of the block size of the LD of the Asian population in the International HapMap II Project (S1 Table) [24, 25].

**Fig 3:**
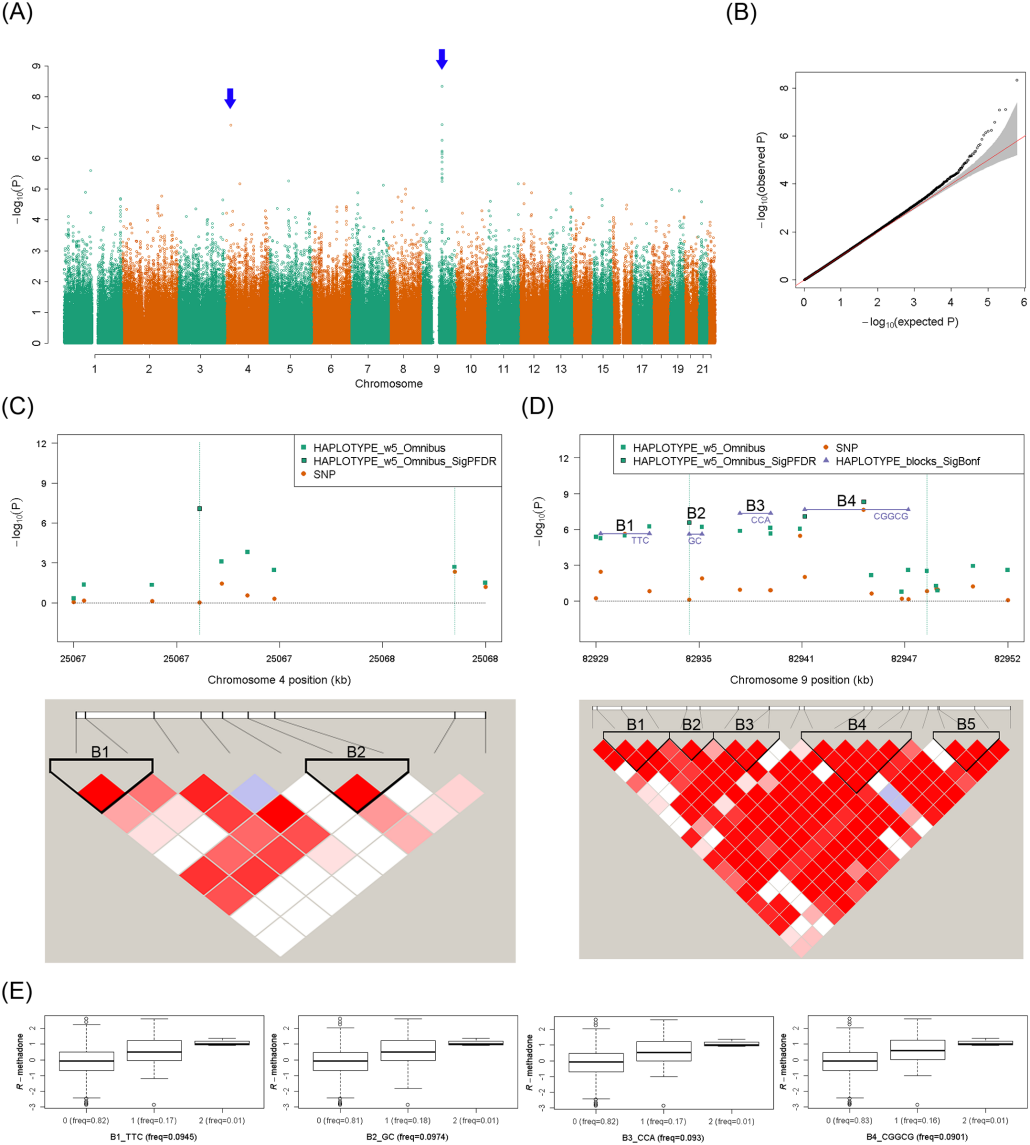
Sliding-window genome-wide haplotype analysis of plasma concentration of *R*-methadone. (**A**) Manhattan plot of genome-wide haplotype-based association test. The vertical axis is the raw *p* values of the omnibus haplotype tests (in a scale of -log10) and the horizontal axis is physical position of the initial SNP of a haplotype in different autosomes. Each point indicates the raw *p* value of a window. The position of a significant haplotype is indicated by a blue arrow. (**B**) Quantile–Quantile plot of genome-wide haplotype-based association test. The vertical axis indicates the raw *p* values of the association tests (in a scale of -log10). The horizontal axis indicates the expected *p* values of the association tests (in a scale of -log10) under a null hypothesis, i.e., no genetic association. (**C**) Regional association plot and LD plot on chromosome 4. In the regional association plot, the vertical axis is the raw *p* values of the association tests (in a scale of -log10) and the horizontal axis is physical position of initial SNP of a haplotype (in unit of kb). Raw *p* values of single SNPs (orange circle) and haplotypes which were significant after adjusting for false discovery rate [26] (symbol: green square with frame) and were not significant (symbol: green square without frame) are shown. Moreover, haplotypes which passed a multiple-test correction of a false discovery rate [26] in a LD block (purple triangle) are shown. In the LD plot, pairwise LD of SNPs was measured by D’ [27]. SNPs with a strong LD were framed in a black inverse diamond block which was defined according to the confidence interval method [24]. (**D**) Regional association plot and LD plot on chromosome 9. (**E**) Distribution of plasma concentration of *R*-methadone for the haplotypes on chromosome 9 significantly associated with the plasma concentration of *R*-methadone.

**Fig 4:**
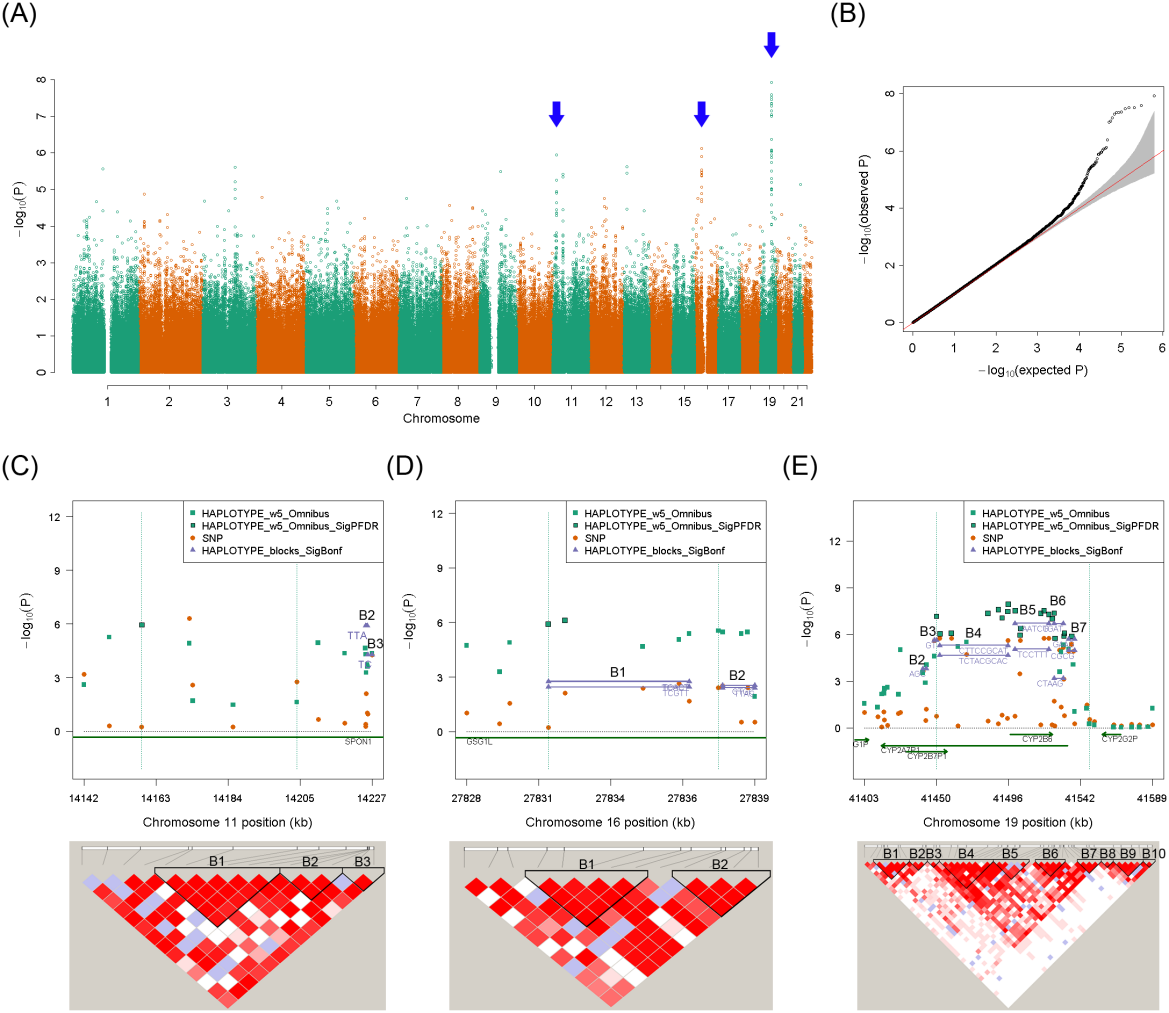
Sliding-window genome-wide haplotype analysis of plasma concentration of *S*-methadone. (**A**) Manhattan plot of genome-wide haplotype-based association test. The vertical axis is the raw *p* values of the omnibus haplotype tests (in a scale of -log10) and the horizontal axis is physical position of the initial SNP of a haplotype in different autosomes. Each point indicates the raw *p* value of a window. The positions of significant haplotypes are indicated by blue arrows. (**B**) Quantile–Quantile plot of genome-wide haplotype-based association test. The vertical axis indicates the raw *p* values of the association tests (in a scale of -log10). The horizontal axis indicates the expected *p* values of the association tests (in a scale of -log10) under a null hypothesis, i.e., no genetic association. (**C**) Regional association plot and LD plot on chromosome 11. In the regional association plot, the vertical axis is the raw *p* values of the association tests (in a scale of -log10) and the horizontal axis is physical position of initial SNP of a haplotype (in unit of kb). Raw *p* values of single SNPs (orange circle) and haplotypes which were significant after adjusting for false discovery rate [26] (symbol: green square with frame) and were not significant (symbol: green square without frame) are shown. Moreover, haplotypes which passed a multiple-test correction of a false discovery rate [26] in a LD block (purple triangle) are shown. In the LD plot, pairwise LD of SNPs was measured by D’ [27]. SNPs with a strong LD were framed in a black inverse diamond block which was defined according to the confidence interval method [24]. (**D**) Regional association plot and LD plot on chromosome 16. (**E**) Regional association plot and LD plot on chromosome 19.

The results for the plasma concentration of *R*-methadone are shown in the Manhattan plot (Fig 3(A)) and Quantile–Quantile plot (Fig 3(B)). The results for the plasma concentration of *S*-methadone are shown in the Manhattan plot (Fig 4(A)) and Quantile–Quantile plot (Fig 4(B)). The results for the plasma concentration of *R*-EDDP (S7(A) Fig) and the plasma concentration of *S*-EDDP (S7(B) Fig) are also provided. After a multiple-test correction of the false discovery rate [26], we determined that four sliding windows were significantly associated with the plasma concentration of *R*-methadone (Fig 3(A)), and 23 sliding windows were associated with the plasma concentration of *S*-methadone (Fig 4(A)). No significant results were found in the haplotype-based association analyses of the plasma concentration of *R*-EDDP (S7(A) Fig) or the plasma concentration of *S*-EDDP (S7(B) Fig).

The four sliding windows associated with the plasma concentration of *R*-methadone are summarized in Table 2; the four windows consisted of one window located on chromosome 4 (Fig 3(C)) and three windows located in consecutive genomic regions on chromosome 9 (Fig 3(D)). The significant window regions were expanded to encompass the flanking region on both sides of the significant windows. The expanded regions on chromosomes 4 and 9 contained two LD blocks (Fig 3(C)) and five LD blocks (Fig 3(D)), respectively. We further examined whether individual haplotypes in the LD blocks were associated with the plasma concentration of *R*-methadone. No significant individual haplotype was found in the significant region of chromosome 4 (Table 2 and Fig 3(C)). Significant individual haplotypes were found in the first four LD blocks in the region of chromosome 9 (Table 2 and Fig 3(D)). The fourth LD block contained the only significant SNP (rs17180299) identified during the genome-wide single-locus association test. The slope estimates and their standard errors and the corresponding 95% confidence intervals of the 4 significant haplotypes are summarized in S2 Table. All of the SNPs were located in intergenic regions. Trends of dose-response effects exerted by the four significant haplotypes on the plasma concentration of *R*-methadone were found (Fig 3(E)). Patients with more minor haplotypes tended to exhibit a higher plasma concentration of *R*-methadone.

**Table 2:** The significant haplotypes associated with plasma concentration of *R*-methadone. We list the chromosome, window, linkage disequilibrium (LD) block, and gene where the haplotypes are located. Haplotype frequencies and raw and adjusted *p* values of the significant haplotypes are provided in the final two columns.

Chrom.	Window ^a^	LD block	Gene	Significant haplotype	Haplotype frequency	Raw *p* value (Adjusted *p*) ^b^
4	w1	1, 2	---	---	---	NS
9	w1-	1	---	*TTC(rs10115245*, *rs62572435*, *and rs1572144)*	0.095	2.26×10^−6^ (4.07×10^−5^)
9	w1	2	---	*GC(rs1333935*, *and rs1333934)*	0.097	2.49×10^−6^ (4.48×10^−5^)
9	w1	3	---	*CCA(rs4085128*, *rs4877551*, *and rs4877552)*	0.093	4.55×10^−8^ (8.19×10^−7^)
9	w2 ~ w3	4	---	*CGGCG(rs970905*, *rs17180299*, *rs12000653*, *rs11138610*, *and rs72744905)*	0.090	2.24×10^−8^ (4.03×10^−7^)

^a^ The sliding-window haplotype-based association analysis using an omnibus association test identified 4 windows associated with the plasma concentrations of *R*-methadone. The number followed by “w” is the index of significant windows. Notation “-” indicates the upstream of a window when we expanded a significant window to encompass the flanking region on either side.

^b^ Notation “NS” indicates the haplotype was not significant.

**Fig 5:**
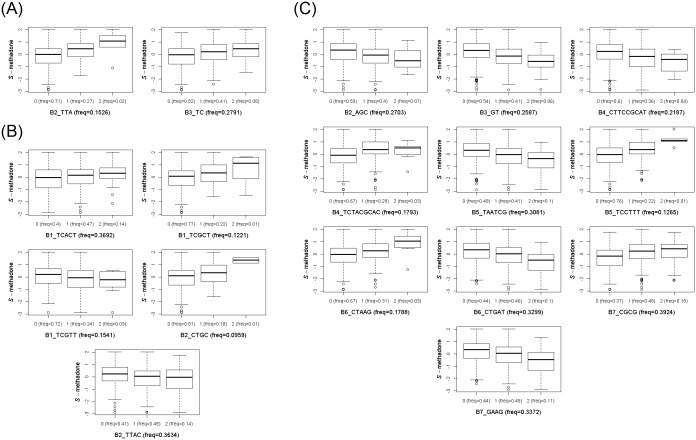
Distribution of plasma concentration of *S*-methadone for the significant haplotypes. Distribution of plasma concentration of each significant haplotype is described in a subfigure; the haplotype frequency is provided in the title. In each subfigure, three box plots for patient groups with respect to 0, 1, and 2 haplotypes of interest are shown. In each box plot, five bands including the 1.5 interquartile range (IQR) of the lower quartile, first quartile, second quartile (i.e., median), third quartile, and 1.5 IQR of the lower quartile of plasma concentration are plotted. The vertical axis is the plasma concentration and the horizontal axis provides haplotype and the proportion of haplotype in our data set. (**A**) Box plots for the significant haplotypes on chromosome 11. (**B**) Box plots for the significant haplotypes on chromosome 16. (**C**) Box plots for the significant haplotypes on chromosome 19.

The 23 sliding windows associated with the plasma concentration of *S*-methadone are summarized in Table 3; the 23 windows were located within three consecutive genomic regions (Fig 4(A)). They contained one window on chromosome 11 (Fig 4(C)), two windows on chromosome 16 (Fig 4(D)), and 20 windows on chromosome 19 (Fig 4(E)). An individual haplotype analysis in LD blocks revealed that 17 haplotypes in the 23 windows were significantly associated with the plasma concentration of *S*-methadone. The slope estimates and the corresponding 95% confidence intervals of the 17 significant haplotypes are summarized in S2 Table. The significant genomic region of chromosome 11 contained two significant haplotypes in LD blocks B2 and B3 on *SPON1* (Table 3 and Fig 4(C)). The significant genomic region of chromosome 16 contained five significant haplotypes in LD blocks B1 and B2 on *GSG1L* (Table 3 and Fig 4(D)). Finally, the significant genomic region of chromosome 19 contained 10 significant haplotypes in LD blocks B2–B7 (Table 3 and Fig 4(E)). This genomic region contained multiple *CYP450* genes. For all the 17 significant haplotypes, trends of dose-response effects exerted by the significant haplotypes on the plasma concentration of *S*-methadone were found; the dose-response effects of the 2, 5, 10 significant haplotypes on chromosomes 11, 16, and 19 are shown in Fig 5(A), 5(B) and 5(C), respectively. S3 and S4 Tables summarize all haplotypes with a minor haplotype frequency of >0.01 whatever they were significant or not in association tests of individual haplotypes for the plasma concentrations of methadone *R*- and *S*-enantiomers, respectively.

**Table 3:** The significant haplotypes associated with plasma concentration of *S*-methadone. We list the chromosome, window, linkage disequilibrium (LD) block, and gene where the haplotypes are located. Haplotype frequencies and *p* values of the significant haplotypes are provided in the final two columns.

Chrom.	Window ^a^	LD block	Gene name	Significant haplotype	Haplotype frequency	Raw *p* value (Adjusted *p*)
11	w1+	2	*SPON1*	*TTA(rs4756776*, *rs4757240*, *and rs4756779)*	0.153	1.27×10^−6^ (1.52×10^−5^)
11	w1+	3	*SPON1*	*TC(rs4757242*, *and rs7936301)*	0.279	5.45×10^−5^ (6.54×10^−4^)
16	w1 ~ w2	1	*GSG1L*	*TCACT(rs60857987*, *rs713547*, *rs705912*, *rs4787995*, *and rs8182215)*	0.368	1.73×10^−3^ (1.38×10^−2^)
16	w1 ~ w2	1	*GSG1L*	*TCGCT(rs60857987*, *rs713547*, *rs705912*, *rs4787995*, *and rs8182215)*	0.122	1.78×10^−3^ (1.42×10^−2^)
16	w1 ~ w2	1	*GSG1L*	*TCGTT(rs60857987*, *rs713547*, *rs705912*, *rs4787995*, *and rs8182215)*	0.154	3.54×10^−3^ (2.83×10^−2^)
16	w2+	2	*GSG1L*	*CTGC(AX-13044173*, *rs772972*, *rs772973*, *and rs11639671)*	0.096	2.96×10^−3^ (2.37×10^−2^)
16	w2+	2	*GSG1L*	*TTAC(AX-13044173*, *rs772972*, *rs772973*, *and rs11639671)*	0.363	3.92×10^−3^ (3.14×10^−2^)
19	w1-	2	*CYP2A7P1*, *CYP2B7P1*	*AGC(rs12461727*, *rs73038469*, *and rs4803406)*	0.270	1.58×10^−4^ (7.11×10^−3^)
19	w1	3	*CYP2A7P1*, *CYP2B7P1*	*GT(rs8110485*, *and rs4124633)*	0.259	2.43×10^−6^ (1.09×10^−4^)
19	w1 ~ w8	4	*CYP2A7P1*, *CYP2B7P1*	*CTTCCGCAT(rs4560022*, *rs3889806*, *rs4803410*, *rs1017384*, *rs7251950*, *rs1808682*, *AX-13442755*, *rs3760657*, *and rs2054675)*	0.219	5.17×10^−6^ (2.33×10^−4^)
19	w1 ~ w8	4	*CYP2A7P1*, *CYP2B7P1*	*TCTACGCAC(rs4560022*, *rs3889806*, *rs4803410*, *rs1017384*, *rs7251950*, *rs1808682*, *AX-13442755*, *rs3760657*, *and rs2054675)*	0.179	2.31×10^−5^ (1.04×10^−3^)
19	w5 ~ w14	5	*CYP2A7P1*, *CYP2B6*	*TAATCG(rs8100458*, *rs7250601*, *rs7250991*, *rs11882424*, *rs8192719*, *and rs10853744)*	0.311	2.03×10^−7^ (9.14×10^−6^)
19	w5 ~ w14	5	*CYP2A7P1*, *CYP2B6*	*TCCTTT(rs8100458*, *rs7250601*, *rs7250991*, *rs11882424*, *rs8192719*, *and rs10853744)*	0.126	9.04×10^−6^ (4.07×10^−4^)
19	w12 ~ w17	6	*CYP2A7P1*	*CTAAG(rs61073883*, *rs1552222*, *rs7255904*, *AX-13442812*, *and rs11666982)*	0.179	6.81×10^−4^ (3.06×10^−2^)
19	w12 ~ w17	6	*CYP2A7P1*	*CTGAT(rs61073883*, *rs1552222*, *rs7255904*, *AX-13442812*, *and rs11666982)*	0.330	2.04×10^−7^ (9.18×10^−6^)
19	w18 ~ w20	7	---	*CGCG(rs17726861*, *rs7257703*, *rs17726963*, *and rs12982859)*	0.392	1.11×10^−5^ (5.00×10^−4^)
19	w18 ~ w20	7	---	*GAAG(rs17726861*, *rs7257703*, *rs17726963*, *and rs12982859)*	0.337	2.08×10^−6^ (9.36×10^−5^)

^a^ The sliding-window haplotype-based association analysis using an omnibus association test identified 23 windows associated with the plasma concentrations of *S*-methadone. The number followed by “w” is the index of significant windows. Notation “-” and “+” indicate the upstream and downstream of a window respectively when we expanded a significant window to encompass the flanking region on either side.

### Analysis of the variation in plasma concentration explained by significant SNP and haplotypes

Table 4 shows the proportions of the variations in the plasma concentrations of methadone *R*- and *S*-enantiomers explained by significant haplotypes. Regarding the plasma concentration of *R*-methadone, one SNP (rs17180299) and four haplotypes on chromosome 9 were significant. Rs17180299 accounted for 9.541% of the variation in the plasma concentration of *R*-methadone. The most significant haplotype, *C-G-G-C-G*, was located in LD block B4 (Fig 3(D)). The SNP rs17180299, which was the only significant SNP identified during the genome-wide single-locus association test, was a tag SNP of the other four SNPs in this region. Therefore, this haplotype accounted for the same amount of variation as did rs17180299. According to the effect exerted by haplotype *C-G-G-C-G*, the remaining three significant haplotypes accounted for an extra variation of 1.809%. In total, 11.350% of the variation in the plasma concentration of *R*-methadone was accounted for by the identified SNPs and haplotypes. Regarding the plasma concentration of *S*-methadone, 17 haplotypes in the 23 windows was included into the final model sequentially; a haplotype which contributed a higher increment of model R^2^ was included earlier in a sequential order. The 17 significant haplotypes accounted for 23.96% of the variation in the plasma concentration of *S*-methadone. The five most significant haplotypes, which included two significant haplotypes in *CYP2B6*, accounted for more than 20% of the variation in the plasma concentration of *S*-methadone.

**Table 4:** The proportion of variation explained by the significant haplotypes we identified. In accordance with the inclusion steps of haplotypes in each analysis of plasma concentration of *R*-methadone and *S*-methadone, we list the chromosome and linkage disequilibrium (LD) block where the haplotypes are located. A haplotype which contributed a higher increment of model R^2^ was included earlier in a sequential order. Univariate R^2^ and model R^2^ of the significant haplotypes are provided in the final two columns.

Transformed plasma concentration	Step	Chrom.	LD block	Significant haplotypes	^a^ Univariate R^2^	^b^ Model R^2^
*R*-methadone	1	9	4	*CGGCG*	0.095408	0.095408
*R*-methadone	2	9	1	*TTC*	0.069709	0.107298
*R*-methadone	3	9	2	*GC*	0.070183	0.113362
*R*-methadone	4	9	3	*CCA*	0.091044	0.113498
*S*-methadone	1	19	5	*TAATCG*	0.07653744	0.075978
*S*-methadone	2	11	2	*TTA*	0.06973389	0.136224
*S*-methadone	3	19	5	*TCCTTT*	0.05598868	0.166829
*S*-methadone	4	16	1	*TCGCT*	0.02923673	0.186557
*S*-methadone	5	16	1	*TCACT*	0.02532389	0.206716
*S*-methadone	6	19	7	*CGCG*	0.05109671	0.21637
*S*-methadone	7	19	2	*AGC*	0.0424036	0.223295
*S*-methadone	8	16	2	*TTAC*	0.02020137	0.230003
*S*-methadone	9	19	3	*GT*	0.0663698	0.233462
*S*-methadone	10	19	4	*TCTACGCAC*	0.0518725	0.235165
*S*-methadone	11	19	6	*CTGAT*	0.07624549	0.236899
*S*-methadone	12	16	1	*TCGTT*	0.02337932	0.238038
*S*-methadone	13	19	6	*CTAAG*	0.03132347	0.238553
*S*-methadone	14	11	3	*TC*	0.04704134	0.239014
*S*-methadone	15	16	2	*CTGC*	0.02666334	0.239358
*S*-methadone	16	19	4	*CTTCCGCAT*	0.0613232	0.239564
*S*-methadone	17	19	7	*GAAG*	0.06556728	0.239564

^a^ Univariate R^2^ indicates the coefficient of determination of the regression model which contains only an intercept term and the single haplotype included.

^b^ Model R^2^ indicates the coefficient of determination of the regression model which contains an intercept term, haplotypes included in previous steps, and the haplotype included in the current step.

### Replication analysis of the significant SNP and haplotypes identified in the genome-wide pharmacogenomic study

S8(A) Fig shows flow charts and results of all statistical analyses, and S8(B) Fig depicts the results of quality control in the replication study. In total, 78 MMT patients were genotyped with the Axiom Genome-Wide CHB 1 Array. Following the same procedures of quality control used at the discovery stage, 76 patients and 613,414 valid SNPs were remained; two (SS-401063 and SS-501002) of 78 patients were removed because of abnormal homozygosity rates. The distributions of the three covariates and two plasma concentrations at the discovery stage and replication stage were similar (Table 1 and S5 Table). The results of association analysis are summarized in S6 Table. Association analysis showed that haplotypes *T-C-G-T-T* on *GSG1L* (raw *p* = 4.40 × 10^−2^), *C-T-T-C-C-G-C-A-T* (raw *p* = 1.90 × 10^−2^) and *T-C-T-A-C-G-C-A-C* (raw *p* = 2.60 × 10^−2^) on *CYP2B7P1* and *CYP2A7P1* were associated with the plasma concentration of *S*-methadone. For clinical applications, we considered urine morphine test, which is an indicator for treatment response to MMT, as a stratified variable. In the test-positive group (i.e., poor-response group), *T*-*T*-*A* (raw *p* = 2.94 × 10^−2^) on *SPON1* was significant; in the test-negative group (i.e., better-response group), *C-T-T-C-C-G-C-A-T* (raw *p* = 3.97 × 10^−2^), *T-C-T-A-C-G-C-A-C* (raw *p* = 3.83 × 10^−2^), and *T-A-A-T-C-G* (raw *p* = 4.37 × 10^−2^) on *CYP2B7P1*, *CYP2A7P1*, and *CYP2B6* were significant. Other haplotypes had a *p* value of >0.05. The five replicated haplotypes (i.e., the significant haplotypes in the 1^st^, 2^nd^, 10^th^, 12^th^, and 16^th^ inclusion steps for the plasma concentration of *S*-methadone in Table 4) accounted for 17.75% and 19.51% of the variation in the plasma concentration of *S*-methadone at the discovery stage and replication stage, respectively. Regression coefficients of the replicated haplotypes at the discovery and replication stages were in the same direction. A meta-analysis by combining *p*-values at the discovery and replication stages showed that most of the haplotypes identified at the discovery stage were still statistically significant (S7 Table). However, we may have overlooked some important findings such as the significance of rs17180299 (raw *p* = 0.3578 in the replication study) and some important haplotypes (S6 Table) because of a small sample size in the replication study.

### Constitutive androstane receptor (CAR)-activated coexpression of *CYP2B6*, *SPON1*, and *GSG1L*

**Fig 6:**
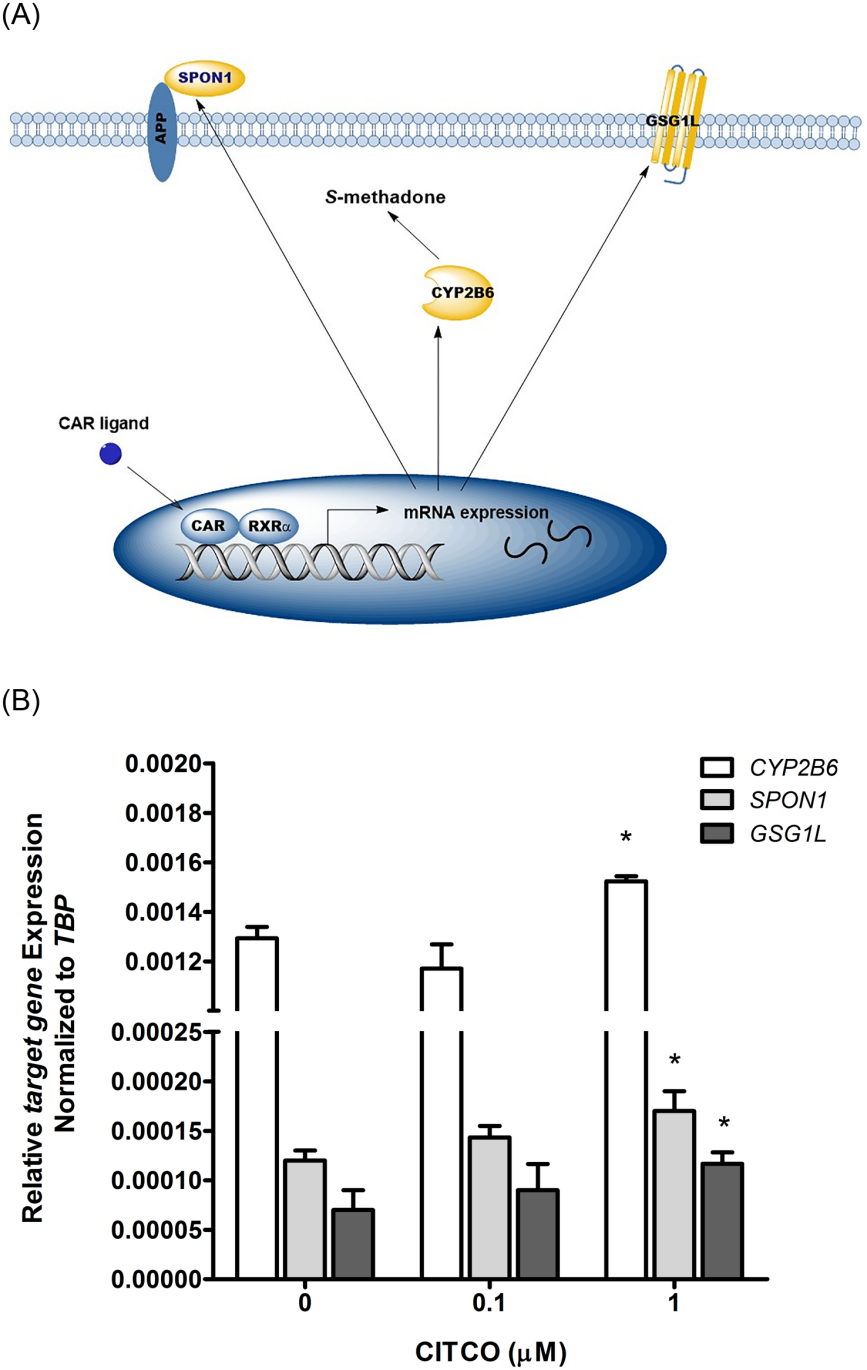
Co-expression of *CYP2B6*, *SPON1*, and *GSG1L* through the CAR activation pathway. (**A**) Schema diagram to show the relation among *CYP2B6*, *SPON1*, and *GSG1L* through the CAR activation pathway. (**B**) Induction of mRNA expression of *CYP2B6*, *SPON1*, and *GSG1L* in HepG2 after a 24-hr treatment with the CAR activator CITCO. In the bar graph, data were presented as the mean of relative mRNA expression ± SD (n = 3). Asterisks above the bars indicate a significant difference in the relative mRNA expression of a gene between a concentration of CITCO of 0 and 1 μM (p < 0.05).

We investigated the roles of *SPON1* and *GSG1L* genes and their coexpression with *CYP2B6* by using a regulated plasma concentration of *S*-methadone in the human liver (Fig 6(A)). The mRNA expression levels of *CYP2B6*, *SPON1*, and *GSG1L* in human hepatoblastoma (HepG2) cells were measured after 24 h of exposure to CAR agonists. The results showed that when the concentration of CAR agonist 6-(4-chlorophenyl)imidazo-[2,1-b][1,3]thiazole-5-carbaldehyde-O-(3,4-dichloro-benzyl)-oxime (CITCO) was increased from 0 μM to 1 μM, the mRNA expression levels of *CYP2B6*, *SPON1*, and *GSG1L* increased concomitantly and significantly (Fig 6(B)). For *CYP2B6*, the mRNA expression increased from 1.293 × 10^−3^ (SD = 4.619 × 10^−5^) to 1.523 × 10^−3^ (SD = 2.082 × 10^−5^), a 1.18-fold increase. For *SPON1*, the mRNA expression increased from 1.200 × 10^−4^ (SD = 1.000 × 10^−5^) to 1.700 × 10^−4^ (SD = 2.000 × 10^−5^), a 1.42-fold increase. For *GSG1L*, the mRNA expression increased from 7.000 × 10^−5^ (SD = 2.000 × 10^−5^) to 1.267 × 10^−4^ (SD = 1.528 × 10^−5^); this was a 1.81-fold increase. The relative gene expression showed that the CAR agonist induced the gene expression of *CYP2B6*, *SPON1*, and *GSG1L*.

## Discussion

*R*- and *S*-methadone can be metabolized through various enzymatic pathways. Results from a previous candidate gene study of *CYP2B6* [16] and other pharmacogenetic studies [10, 11] have shown that the methadone *S*-enantiomer was frequently metabolized by CYP2B6 isozymes. The present genome-wide association study confirmed that *CYP2B6* is associated with the plasma concentration of methadone *S*-enantiomer. The two significant haplotypes, *T-A-A-T-C-G* and *T-C-C-T-T-T* of rs8100458, rs7250601, rs7250991, rs11882424, rs8192719, and rs10853744, on *CYP2B6* that were identified in this study accounted for 10.72% of the variation in the plasma concentration of *S*-methadone. Haplotype *T-C-C-T-T-T* was found to be associated with a urine morphine test in a permutation-based logistic regression analysis with 100,000 random shuffles of test-positive and test-negative patients (empirical *p* = 4.04 × 10^−3^). The results implied that the identified haplotypes were relevant to the response to MMT.

We further examined the genetic contribution of *CYP2B6* by performing an integrative analysis that involved seven SNPs (rs8100458, rs7250601, rs7250991, rs11882424, rs8192719, rs10853744, and rs1042389) on *CYP2B6* from the present study and 10 SNPs (rs8100458, rs10500282, rs10403955, rs2279342, rs3745274, rs2279343, rs2279345, rs1038376, rs707265, and rs1042389) from a previous study of *CYP2B6* [16]. Overall, the haplotype analysis involved 15 distinct SNPs, including two SNPs (rs8100458 and rs1042389) that were investigated in the two studies. LD blocks were constructed and haplotype association tests were conducted in each LD block by using the same association test described in the **Methods** section. The analysis revealed two LD blocks (S9 Fig). The block near the 3’ end was an untranslated region and contained three SNPs (rs1038376, rs707265, and rs1042389 [i.e., AX-13442801]). Haplotypes *A*-*A*-*T* (raw *p* = 2.25 × 10^−7^, haplotype frequency = 0.332) and *T*-*G*-*T* (raw *p* = 5.64 × 10^−4^, haplotype frequency = 0.142) were significantly associated with the plasma concentration of the methadone *S*-enantiomer. The block near the 5’ end contained 12 SNPs. Haplotypes *T-A-A-T-T-A-G-G-T-T-C-G* (raw *p* = 1.10 × 10^−3^, haplotype frequency = 0.024), *T-A-A-T-T-A-G-A-T-T-C-G* (raw *p* = 1.65 × 10^−4^, haplotype frequency = 0.278), and *T-C-C-C-G-A-T-G-C-T-T-T* (raw *p* = 7.04 × 10^−6^, haplotype frequency = 0.126) were significant.

The results of the two haplotype analyses from the current study and the aforementioned combined analysis of 15 SNPs were compared. Two significant haplotypes of six SNPs on *CYP2B6* identified in the current study were also in the LD block near the 5’ end. The patterns of these two haplotypes were compatible with the three significant haplotypes identified in the combined analysis of 15 SNPs. The two significant haplotypes can be presented as *T-A-A-X-X-X-X-X-X-T-X-C-G* (raw *p* = 2.03 × 10^−7^, haplotype frequency = 0.311) and *T-C-C-X-X-X-X-X-X-T-X-T-T* (raw *p* = 9.04 × 10^−6^, haplotype frequency = 0.126), where *X* denotes an unknown allele caused by the unavailability of the SNPs in the Axiom Genome-Wide CHB 1 Array. The proportion of the variation in the plasma concentration of methadone *S*-enantiomers accounted for by the five haplotypes on *CYP2B6* increased from 10.725% to 13.433% after the SNPs of the Axiom Genome-Wide CHB 1 Array and the SNPs from our previous candidate gene study of *CYP2B6* were combined. Of the 15 SNPs, two were missense SNPs: rs3745274 (raw *p* = 8.08 × 10^−7^, minor allele frequency = 0.185) and rs2279343 (raw *p* = 3.62×10^−3^, minor allele frequency = 0.263). These two SNPs have been associated with methadone in heroin-dependent patients [12] and poor metabolism, plasma concentration, and efavirenz clearance in children with human immunodeficiency virus [28–30].

*CYP2B6* was highly expressed in the liver (S10(A) Fig) and influenced methadone metabolism. We investigated whether the identified haplotypes near *CYP2B6* can regulate the gene expression of *CYP2B6* through the mechanism of cis-regulation. An RT-PCR experiment was conducted to measure the gene expression of *CYP2B6* in a random sample of 55 patients. The results showed that none of the 10 haplotypes associated with the plasma concentration of methadone *S*-enantiomer in the third significant region of chromosome 19 (Fig 4(E)) achieved significance. Only haplotype *A*-*G*-*C* of rs12461727, rs73038469, and rs4803406 in LD block B2 in *CYP2A7P1* and *CYP2B7P1* showed a dose-response pattern and had a *p* value near a marginal significance level (raw *p* = 0.0644). More samples are needed to verify that haplotype *A*-*G*-*C* is an expression quantitative trait locus.

In addition to the association between the *CYP2B6* gene and the plasma concentration of methadone *S*-enantiomers, we discovered that the haplotype SNPs located in the gene regions of *SPON1* and *GSG1L* were significantly associated with the plasma concentration of methadone *S*-enantiomers. To investigate the roles of *SPON1* and *GSG1L* genes and their coexpression with *CYP2B6* in a regulated plasma concentration of *S*-methadone in the liver, the cultured human liver cell line HepG2 was incubated with the CAR agonist CITCO, a CYP2B6 inducer [31]. The mRNA expression levels of *CYP2B6*, *SPON1*, and *GSG1L* in HepG2 cells were measured; the results revealed that the gene expression of *CYP2B6*, *SPON1*, and *GSG1L* can be activated concomitantly through the CAR activation pathway. Methadone reportedly activates the CAR [32], a phenomenon that partially explains why the three genes were significantly associated with the plasma concentrations of *S*-methadone. *SPON1* was expressed in all the tissues, including the brain, lymph node, kidney, and intestine (S10(B) Fig). *GSG1L* was expressed mostly in connective tissue and the tissues of the brain, eye, and testis (S10(C) Fig). Both *SPON1* and *GSG1L* were expressed in brain tissue. A previous study found that *GSG1L* was an α-amino-3-hydroxy-5-methyl-4-isoxazolepropionic acid (AMPA) glutamate receptor relevant to neurotransmission [33]. In the present study, a permutation-based association test with 100,000 random shuffles was conducted, revealing that haplotype *C-T-G-C* was associated with treatment emergent symptoms for adverse events related to MMT (empirical *p* = 1.82 × 10^−2^). In addition, the preliminary interaction analysis suggested that there was an interactive effect between haplotype *T*-*C* in *SPON1* and haplotype *T*-*C*-*G*-*C*-*T* in *GSG1L* (raw *p* = 7.55 × 10^−3^). According to these findings, the roles of *SPON1* and *GSG1L* in the brain and the methadone action pathway will be further investigated in future studies.

The methadone *R*-enantiomer reportedly tends to be metabolized by the CYP2C19 isozyme [8, 9]. Our previous study found that a genotype combination of two functional SNPs (rs4986893 in exon 4 and rs4244285 in exon 5) on *CYP2C19* were associated with the plasma concentration of methadone *R*-enantiomers (*p* = 0.007; the *p* values of rs4986893 and rs4244285 were 0.8 and 0.013, respectively). Moreover, the genotype combination can provide a gene dosage model for classifying poor, intermediate, and extensive metabolizers [34]. On the basis of the same dataset, the current analysis further revealed that rs4986893, rs4244285, and their genotype combination accounted for 0.198%, 2.014%, and 2.351% of the variation in the plasma concentration of methadone *R*-enantiomer, respectively.

This genome-wide single-locus association analysis did not find a single SNP on *CYP2C19* that exceeded the genome-wide significance level in the association analysis of the plasma concentration of methadone *R*-enantiomer. In addition, rs4244285 on *CYP2C19* was not designed in the Axiom CHB arrays. In contrast to the candidate gene study, this genome-wide single-locus association analysis identified that only the SNP rs17180299 (raw *p* = 2.24 × 10^−8^) had a strong signal of genetic association. Patients with more allele *G* of rs17180299 on average had higher plasma concentration of methadone *R*-enantiomer. Rs17180299, which accounted for 9.541% of the variation in the plasma concentration of methadone *R*-enantiomer, accounted for a substantially greater variation than did rs4986893, rs4244285, and their genotype combination. This SNP may be crucial for predicting the plasma concentration of methadone *R*-enantiomer and classifying heroin-dependent patients with different methadone metabolism responses. Permutation-based association tests with 100,000 random shuffles further revealed that haplotype *C-G-G-C-G* (Fig 3(D)), which contained rs17180299, was associated with the weight-adjusted methadone dosage (empirical *p* = 2.46 × 10^−2^). Previous studies have suggested that methadone *R*-enantiomers induce an enhanced analgesic effect; however, it also exerts an adverse and toxic cardiac effect [35, 36]. Therefore, patients with more allele *G* can be administered lower doses of methadone *R*-enantiomers to minimize the adverse effect.

Although rs17180299 is located in an intergenic region, the data from the Roadmap Epigenomics Projects revealed a relationship between rs17180299 and heterochromatic histone H3 lysine 9 trimethylation (H3K9me3) in the primary T regulatory cells from peripheral blood (*p*-value signal score was 2.47354). Several studies reported the association of histone mark H3K9me3 and substance abuse. For example, cocaine dynamically regulates H3K9me3 [37] and H3K9me3 is a key player in the regulation of affective vulnerability and substance abuse [38]. The information suggests that rs17180299 may play a role in the regulation of plasma concentration of methadone *R*-enantiomer through epigenetic histone modification of H3K9me3.

We also imputed untyped SNPs using the SHAPEIT software [39] and IMPUTE2 software [40] and ran association analysis in a 2-Mb flanking region of rs17180299 on chromosome 9. Strong association signals were found in the almost complete LD region closest to rs17180299 (S11 Fig). Among the significant SNPs, the imputed SNP rs17083093 at 82,938,858 bp (Raw *p* = 6.80 ×10^−9^) was even more significant than rs17180299 at 82,944,201 bp (Raw *p* = 2.24 ×10^−8^). All of the significant SNPs were located in intergenic regions and only rs17180299 provides direct biological explanations for substance abuse.

In general, the available sample sizes in pharmacogenomic studies are smaller than the sample sizes in genome-wide association studies for complex diseases. In this MMT studies, most patients were not willing to come to the clinic in daily basis for more than 5 days to fulfil the requirement of the steady-state status in which the half-life of methadone was considered with an average of 24 hours. This study overcame the difficulty and recruited a moderate sample size of steady-state MMT patients through multicenter collaborations. Furthermore, we successfully replicated that *CYP2B6*, *SPON1*, and *GSG1L* are associated with the plasma concentration of methadone *S*-enantiomer. However, we may have overlooked some important findings such as the significance of rs17180299 and some important haplotypes because of a small sample size in the replication study. SNP rs17180299 cannot be replicated because of a low frequency of minor allele *G* in the replication study; several MMT patients with genotype *GG* were observed at the discovery stage but no patients with genotype *GG* were observed at the replication stage. We calculated the sample size required to replicate the association of significant individual haplotypes by using Quanto software [41]. For significant individual haplotypes with a stronger effect (e.g., the haplotypes on *CYP2B6*), on the basis of the current sample size, we had a power of 0.65 to replicate the results. For the haplotypes with a weaker effect (e.g., the haplotypes on *GSG1L*), on the basis of the current sample size, we had only a power of 0.25 to replicate the results. In the latter situation, we will need 330 replication samples in order to attain a power of 0.80. On the other hand, the replication of the association findings was mainly based on haplotype-specific association tests. Further investigation should be undertaken to prevent spurious association findings. We also evaluated the influence of “winner’s curse” on the estimation of the proportion of variation explained by the five replicated haplotypes. The replicated haplotypes accounted for 17.75% and 19.51% of the variation in the plasma concentration of *S*-methadone at the discovery stage and replication stage, respectively. Because the two proportions of variation explained were close, this information suggested no winner’s curse in this study. In this study, we examined samples from a single East-Asian population to reduce false-positive and false-negative results caused by genetic heterogeneity. Currently, no other genome-wide pharmacogenomic study on the plasma concentration of methadone in other populations exists for comparison.

In conclusion, this study was the first genome-wide pharmacogenomic study to identify genes associated with the plasma concentrations of methadone *R*- and *S*-enantiomers and their respective metabolites in a methadone maintenance cohort of heroin-dependent patients. A significant intergenic SNP (rs17180299) and four intergenic haplotypes on chromosome 9 accounted for 11.350% of the variation in the overall plasma concentration of methadone *R*-enantiomers. Regarding the plasma concentration of methadone *S*-enantiomers, 17 significant haplotypes were identified: two haplotypes on *SPON1*, five haplotypes on *GSG1L*, and 10 haplotypes on *CYP450* genes, including *CYP2B6*. These haplotypes accounted for approximately one-fourth of the variation in the plasma concentration of *S*-methadone. These results revealed that additional candidate genes are associated with the plasma concentration of methadone, affording insights into the mechanism of methadone metabolism, and facilitating further enhancement of MMT.

## Materials and Methods

### Ethics statement

Written informed consent was obtained from all participants during their initial clinical visit. The genome-wide pharmacogenomic study was approved by the Internal Review Boards of National Health Research Institutes (Zhunan, Taiwan; Permit Number: EC0970504) and the seven participating clinical centers, Taoyuan Mental Hospital, En-Chu-Kong Hospital, Far-Eastern Memorial Hospital, Taipei City Hospital Song-De Campus, China Medical University and Hospital, Taipei City Hospital Yang-Ming Campus, and Wei Gong Memorial Hospital (Miaoli, Taiwan). In addition, this study was registered with the U.S. National Institutes of Health Clinical Trial Registry (http://www.clinicaltrial.gov/ct/show/NCT01059747). The replication study was approved by the Institutional Review Boards of China Medical University (Taichung, Taiwan; Permit Number: DMR101-IRB1-218 EC0970504).

### Study participants

This genome-wide pharmacogenomic study recruited 360 heroin-dependent patients who underwent the MMT. The inclusion and exclusion criteria for participation are described as follows. This study included patients who (1) resided in Taiwan and were Han Chinese; (2) were older than 18 years; (3) could participate in a clinical assessment in Chinese (including Mandarin and Taiwanese dialects); (4) were diagnosed as a patient with heroin dependence based on the Diagnostic and Statistical Manual of Mental Disorders, Fourth Edition; (5) underwent the MMT for at least 3 months; (6) received the MMT regularly in the past one week; (7) had no change of >10 mg in methadone dosage throughout the past one week; and (8) signed the informed consent form. This study excluded patients (1) who were pregnant and (2) who had severe cognitive impairment or severe comorbid mental disorders, including organic mental disorders and schizophrenia. For replication, we recruited another 78 independent samples under the same inclusion and exclusion criteria of the genome-wide pharmacogenomic study.

### Methadone and its metabolites in the plasma

The plasma concentrations of methadones and its metabolite EDDP enantiomers were measured using high-performance liquid chromatography according to the settings described in our previous report [41]. A Chiral-AGP analytical column was used (5 μm, 100 × 3 mm; Chrom Tech, Cheshire, UK) with a Chiral-AGP guard column (10 × 3 mm) (Chrom Tech). Methadone, EDDP, and amitriptyline as an internal standard (40 ng) were extracted from the plasma samples by using a C18-E Strata solid-phase extraction column with a 100-mg/mL capacity (Phenomenex, Torrance, CA). In addition, 800-μL aliquots of each serum sample and 40 ng of the amitriptyline internal standard were added. The column was then washed and the remaining compounds were eluted. The collected eluent was then evaporated and the remaining residue was dissolved in 100 μL of mobile phase. A total sample volume of 50 μL was chromatographed. The intraday and interday coefficients of variation were 3.3% and 6.6% for *R*-methadone, 2.5% and 5.6% for *S*-methadone, 1.6% and 3.9% for *R*-EDDP, and 2.8% and 5.5% for *S*-EDDP, respectively. The recovery rates for *R*-methadone, *S*-methadone, *R*-EDDP, and *S*-EDDP were 109.0 ± 7.6%, 96.7 ± 8.6%, 96.6 ± 6.6%, and 87.4 ± 3.2%, respectively. The recovery rate for the internal standard was 60.2 ± 4.8%. The details are described in our previous publications [42, 43].

### DNA samples and SNP genotyping experiments

The genomic DNA of 360 Han Chinese MMT patients at discovery stage and 78 Han Chinese MMT patients at replication stage were isolated from leukocytes by using a Puregene Blood kit C (QIAGEN Sciences, Germantown, Maryland). The DNA concentration was measured and adjusted to 15–20 ng/μL by using a NanoDrop ND-1000 Spectrophotometer (NanoDrop Technologies, DE). All samples were genotyped at the National Center of Genomic Medicine (Taipei, Taiwan) by using the Axiom Genome-Wide CHB 1 Array (Affymetrix, Inc., San Diego, CA) following the manufacturer’s protocols (http://www.affymetrix.com). The genotype calling was performed using the Genotyping Console 4.0 with default parameters (http://www.affymetrix.com). This SNP genotyping platform optimized the genomic coverage of common SNPs having a minor allele frequency of >5% of the genomes of Han Chinese populations. In total, this array comprised 642,832 SNPs selected from the Axiom Genomic Database, which includes SNP probes from the International HapMap Project (http://hapmap.ncbi.nlm.nih.gov/), the 1,000 Genomes Project (http://www.1000genomes.org/), and the dbSNP database (http://www.ncbi.nlm.nih.gov/SNP/). Based on the NetAffyx Annotation Version 32.1, the SNPs included 625,360 autosomal SNPs, 14,342 nonautosomal SNPs, 59 SNPs without chromosome information, and 3,071 control SNPs.

### Preparation of liver cell lines and real-time polymerase chain reaction (RT-PCR) analysis

CAR agonist CITCO was purchased from Sigma-Aldrich (St. Louis, MO). CITCO (1 mM) was dissolved in 100% dimethyl sulphoxide (DMSO) and diluted with 10 times as much culture media (final DMSO concentration of 0.1%). HepG2 cells (Passage 96) were obtained from the Culture Collection and Research Centre (Food Industry Research and Development Institute, Hsinchu, Taiwan). The cells were cultured in Eagle’s Minimum Essential Medium supplemented with 10% fetal bovine serum (FBS) (Biological Industries, Kibbutz Beit Haemek, Israel). The cultures were maintained in a humidified atmosphere with 5% CO_2_ at 37°C, and the medium was refreshed every three or four days.

The HepG2 cells (Passages 98–100) were seeded on 10-cm petri dishes with a density of 2.5 × 10^5^ cells/10 mL of culture medium supplemented with 10% FBS. After 24 h of overnight cellular attachment, the medium was replaced with a medium containing different concentrations of CITCO or a vehicle (0.1% DMSO). The drug was incubated with the cells for 24 h. The entire cellular RNA was isolated using a Trizol reagent according to the manufacturer’s protocol (Life Technologies, Carlsbad, CA).

RT-PCR was conducted using a RevertAid H Minus First Strand cDNA Synthesis Kit (Fermentas, Waltham, MA) featuring a random hexamer and the RT-PCR operating on an ABI StepOne Plus System. The RT-PCR was performed for *CYP2B6*, *SPON1*, *GSG1L*, and a housekeeping gene, TATA-box binding protein (*TBP*), by using predesigned gene-specific TaqMan probes and primer sets (Hs03044634_m1 for *CYP2B6*, Hs01120488_m1 for *SPON1*, Hs00896151_m1 for *GSG1L*, and Hs00920497_m1 for *TBP*) purchased from Applied Biosystems (Applied Biosystems, Foster City, CA). Gene expression was quantified relative to *TBP* expression by using ABI StepOne Plus Software. The relative expression level of *CYP2B6*, *SPON1*, or *GSG1L* compared with that of *TBP* was defined as CT = [CT_target gene_CT_*TBP*_], where CT is the cycle threshold. The ratio of the mRNA of a target gene to the mRNA of *TBP* was calculated by using 2 ^CT^. The relative expression level of *CYP2B6*, *SPON1*, or *GSG1L* within various concentrations of the CITCO was calculated and compared according to an unpaired Student’s *t* test by using GraphPAD Prism, Version 5 (GraphPad Software, Inc., La Jolla, CA). Data were represented as mean ± SD of triplicates of the experiment.

### Statistical analyses

Fig 1(A) depicts a flowchart of the overall statistical analyses. Quality of samples and SNPs were controlled, and genome-wide single-locus association tests and haplotype association tests were performed. The identified SNPs, haplotypes, and genes were further examined through a replication study. The details of analysis procedures are described below.

The quality of samples and SNPs was examined according to the procedures in the analysis protocol [44] in addition to examinations developed in this study. Fig 1(B) shows a summary of the procedures and results. Prior to examination, 3,071 control SNPs provided in the Axiom Genome-Wide CHB 1 Array were removed. Regarding sample quality control, first, we assessed the concordance of the sex information according to the self-reported gender and homozygosity pattern on X chromosomes; male participants with a homozygosity rate of <0.2 and female participants with a homozygosity rate of >0.8 were identified as having a sex error, and the homozygosity pattern of their X chromosomes was further examined. After the sex examination, we excluded 14,342 nonautosomal SNPs from the subsequent analyses. Second, we calculated the GCR; patients who had a GCR of <0.95 were identified as poor-quality samples. Third, the average genome-wide homozygosity rate was calculated and the 99.7% confidence interval was constructed; patients who had a genome-wide homozygosity rate outside the confidence interval were identified as outlying patients, and the pattern of their run of homozygosity was further examined. Fourth, we identified patients with unknown familial relationships; the IBD of each pair of individuals was estimated, and the pairs of individuals who had an estimated IBD of >0.1875 (i.e., greater than the average expected IBD values for second- and third-degree relatives) were identified as having cryptic relatedness. Finally, we identified individuals who showed evidence of divergent ancestry; the confidence bands of the first two principal components of allele frequency were constructed for each of five populations, including the Han Chinese population in Taiwan and four additional populations from the International HapMap II Project. The patients from Taiwan whose values of the first two principal components were located outside the confidence bands of the Han Chinese population in Taiwan were regarded as having divergent ancestry.

Regarding SNP quality, we first calculated the GCR of the SNPs; the SNPs that had a GCR of <0.95 were identified as poor-quality SNPs. Second, the MAF was estimated using an allele counting approach; the SNPs that had an MAF of <0.01 were identified as nonpolymorphic markers. We examined the HWE by using an exact HWE test [45]; SNPs that had a *p* value that was less than the Bonferroni genome-wide significance level were identified as deviations from the HWE. Finally, after the SNPs associated with the four quantitative traits of study were identified, we examined the SNP cluster plots to ensure that the genotype calls were reliable.

In the aforementioned quality control of samples and SNPs, the patterns of homozygosity on X chromosomes and excessive runs of homozygosity in the human genome were examined using LOHAS software [46]. The R programs in this study were used to construct principal components of allele frequency and their confidence bands and identify patients with divergent ancestry. Other statistical quality control procedures were performed using PLINK software [47].

Four quantitative traits (i.e., plasma concentrations of the methadone *R*- and *S*-enantiomers and their metabolites) were examined in the genetic association analysis. Before the genetic association analysis, data transformation to normality was performed as follows: the quantitative traits were transformed through a winsorization procedure with a threshold of 0.01, followed by a log transformation, and then standardized by subtracting the mean from the traits and dividing the results by the standard deviation. Normality of the transformed data was tested by a Kolmogorov–Smirnov goodness-of-fit test [23]. The aforementioned data transformation was completed using R programs.

The normalized quantitative traits were studied through genetic association analyses, including the genome-wide single-locus association test and haplotype association test. The genome-wide single-locus association test was performed according to a linear regression model by using PLINK software [47]. The four normalized quantitative traits were individually considered as dependent variables. The independent variables included a SNP variable coded as 0, 1, and 2 for genotypes *AA*, *Aa*, and *aa*, respectively, and three additional variables for covariate adjustments, including age, sex, and BMI. The *p* value of a one degree of freedom Wald test was obtained for each SNP to examine the additive genetic effect. During each analysis of the four quantitative traits, the *p* values on a scale of -log_10_ obtained from genome-wise single-locus association tests were displayed in a Manhattan plot and a Quantile–Quantile plot. The false discovery rate [48] was determined for a multiple-test correction. Finally, the significant SNPs were depicted in a Regional Association Plot [49].

We performed genome-wide sliding-window haplotype-based association analyses with different window sizes individually. The human genome was partitioned into a series of sliding windows according to a specific window size. For each sliding window, a four-step haplotype analysis was performed as follows: First, haplotype configurations were inferred using a standard expectation-maximization algorithm [50]. Only haplotype configurations with a posterior probability of >0.01 and only haplotypes with a minor haplotype frequency of >0.01 were analyzed in the subsequent steps. Second, an omnibus haplotype association test with linear regression analysis with an adjustment for age, sex, and BMI was performed using PLINK software. The omnibus test, which involved a Wald test with a degree of freedom of *h*-1 (where *h* is the number of distinct haplotypes in the window), was used to examine whether all haplotypes in the study window were associated with an overall study trait.

Third, a window that was significant in the second step was expanded to encompass the flanking region on either side. The width of extension on each side was one half of the window width (i.e., the physical position of the last SNP minus the position of the first SNP in the window). If windows overlapped, they were combined to form a new window before the region was expanded. Finally, in each extended genomic region defined at the third step, LD blocks were constructed based on the Gabriel’s method [24] by using HAPLOVIEW [51]. In each LD block, a linear regression analysis with an adjustment for age, sex, and BMI was conducted using PLINK to examine the association between the study traits and individual haplotypes versus that of other haplotypes. All haplotypes with a minor haplotype frequency of >0.01 were analyzed and the raw *p* values of individual haplotypes were adjusted for multiple testing by using the false discovery rate in the entire extended regions of the significant windows and also the Bonferroni correction by the extended regions of the significant windows. Only the haplotypes which passed both of the corrections were claimed to be significant.

For each of the studied quantitative traits, the proportion of variation accounted for by the significant SNPs and haplotypes were calculated as follows. Based on the variable(s) or covariate(s) in a regression model, the next SNP or haplotype was included if the SNP or haplotype produced the maximal increment of model R^2^. Using this variable inclusion procedure, we included all SNPs and haplotypes significantly associated with a quantitative trait in the regression model sequentially. Model R^2^ revealed the coefficient of determination of a full regression model that contained one or more haplotypes. In addition, the marginal R^2^ was calculated for each SNP or haplotype according to the regression model that contained only that SNP or haplotype.

In a replication study, we evaluated quality control of samples and SNPs using the same quality control procedures at the discovery stage. The identified SNPs and haplotypes in the genome-wide pharmacogenomic study were further examined using the same genetic association tests at the discovery stage. A meta-analysis by combining *p*-values at the discovery and replication stages was performed by using Fisher’s product *p*-value method [52]. S8(A) Fig depicts a flowchart and results of the statistical analyses and S8(B) Fig shows a summary of the procedures and results of quality control of samples and SNPs in the replication study.

## Supporting Information

S1 Table Distributions of windows with different sizes and distributions of LD blocks with different sizes. In terminology, the window width indicates the physical distance between the first and last SNPs in a window, and the window size indicates the number of SNPs in a window. For each window size of 2, 3, 4, 5, and 10 SNPs, the frequency of windows with an average width of 0–1 kb, 1–10 kb, 10–20 kb, 20–50 kb, and >50 kb in our study are provided. In the last column, the frequency of linkage disequilibrium (LD) blocks with an average width of 0–1 kb, 1–10 kb, 10–20 kb, 20–50 kb, and >50 kb in the HapMap Asian population are provided [24, 25].
(DOCX)

S2 Table Effect sizes of the significant haplotypes we identified. In accordance with the inclusion steps of haplotypes in each analysis of plasma concentration of *R*-methadone and *S*-methadone, we list the chromosome and linkage disequilibrium (LD) block where the haplotypes are located. Slope estimate and its standard error (s.e.) and the corresponding 95% confidence interval are provided in the final two columns.
(DOCX)

S3 Table All haplotypes in association tests of individual haplotypes for plasma concentration of *R*-methadone. We list the chromosome, window, linkage disequilibrium (LD) block, and gene where the haplotypes are located. Haplotype frequencies and raw and adjusted *p* values of the significant haplotypes are provided in the final two columns.
(DOCX)

S4 Table All haplotypes in association tests of individual haplotypes for plasma concentration of *S*-methadone. We list the chromosome, window, linkage disequilibrium (LD) block, and gene where the haplotypes are located. Haplotype frequencies and raw and adjusted *p* values of the significant haplotypes are provided in the final two columns.
(DOCX)

S5 Table Descriptive statistics of three covariates and two quantitative traits. The number of individuals and means ± standard deviations (SD) of covariates and quantitative traits are provided by gender. In the final column, *p* values of the Kolmogorov-Smirnov Good-of-Fit tests for normality for the pre- and post-transformation data of four quantitative traits.
(DOCX)

S6 Table Haplotype frequencies and *p*-values of the significant haplotypes, identified by our genome-wide pharmacogenomic study, at the discovery and replication stages. We list the chromosome (Chrom.), linkage disequilibrium (LD) block, and significant haplotypes followed by their haplotype frequencies (HF) and raw *p*-values (P) at discovery stage and replication stage. The results at the replication stage are further stratified according to the urine morphine test (UMT): UMT = All, Negative, and Positive.
(DOCX)

S7 Table Results of a meta-analysis by combining *p*-values at the discovery and replication stages. We list the chromosome (Chrom.), linkage disequilibrium (LD) block, and significant haplotypes followed by their *p*-values according to the urine morphine test (UMT): UMT = All, Negative, and Positive.
(DOCX)

S1 Fig Sex check plots. The homozygosity intensities of each individual are calculated and plotted based on 12,531 SNPs on chromosome X using software LOHAS. Five individuals exhibited a homozygosity pattern of X chromosome(s) inconsistent to their self-reported gender: (**A**) Individual 206–055: the self-report gender is “female” but the pattern of homozygosity intensity suggests that chromosome X is hemizygous or homozygous. (**B**) Individual 301–041: the self-report gender is “female” but the pattern of homozygosity intensity suggests that chromosome X is hemizygous or homozygous. (**C**) Individual 306–023: the self-report gender is “female” but the pattern of homozygosity intensity suggests that chromosome X is hemizygous or homozygous. (**D**) Individual 306–026: the self-report gender is “male” but the pattern of homozygosity intensity suggests that chromosome X is neither hemizygous nor homozygous. (**E**) Individual 401–023: the self-report gender is “male” but the pattern of homozygosity intensity suggests that chromosome X is neither hemizygous nor homozygous.
(TIF)

S2 Fig Homozygosity check plot. The genome-wide homozygosity rate (i.e., one minus heterozygosity rate) and proportion of missing genotype (i.e., one minus genotyping call rate) of each individual are calculated and plotted based on autosomal SNPs. The two vertical reference lines are the mean homozygosity rate ± 3 standard deviations. Four individuals are located outside the lower and upper reference lines of homozygosity rate, including three samples (217–024, 301–156, and 306–013) with an over-high genome-wide homozygosity rate and one sample (206–059) with an over-low genome-wide homozygosity rate are found. The sample (206–059) was already removed because of a low genotyping call rate of <0.95.
(TIF)

S3 Fig Two outlier individuals (217–024 and 301–156) who carried multiple excessive runs of homozygosity on multiple chromosomes. (**A**) Principal component plot of homozygosity intensity identified two outlier MMT patients (217–024 and 301–156) who carried multiple excessive runs of homozygosity on multiple chromosomes. (**B**) For patient 217–024, chromosome-wise homozygosity intensity plot found excessive runs of homozygosity on chromosome 1, 2, 5, 7, 11 and 15. (**C**) For patient 301–156, chromosome-wise homozygosity intensity plot found excessive runs of homozygosity on chromosome 5. Each of (B) and (C) consists of 22 subfigures. Each subfigure presents a homozygosity intensity plot for one autosomal chromosome. The vertical axis is the estimated homozygosity intensity, and the horizontal axis is physical position (Mb). Each point denotes an anchor SNP of a sliding window, and the gap in each subplot represents the centromeric gap. The reference line is a homozygosity intensity threshold of 0.9.
(TIF)

S4 Fig Principal component plot of allele frequency. Principal component plot of 344 MMT patients in Taiwan (MMT; pink color) and 210 independent individuals from the International HapMap II Project [53]. The HapMap II individuals consisted of 30 married African couples from Yoruba in Ibadan (YRI; green color), 30 married Caucasian couples of European-descent residing in Utah (CEU; cyan color), and 90 Asian people, including 45 Han Chinese people in Beijing (CHB; orange color) and 45 Japanese people in Tokyo (JPT; purple color). The ellipse curves indicate the 99.99% confidence bands of the first two principal components of allele frequency. The individuals from African, European and Asian populations are well classified in the left-hand figure. The right-hand figure zooms in the cluster pattern of three East-Asian populations. In comparison, the MMT patients from Taiwan are closer to the CHB people than the JPT people. Two MMT patients (sample ID: 301–011 and 301–107) lie outside the confidence band and identified as divergent ancestry outliers of the MMT patients in Taiwan.
(TIF)

S5 Fig Distributions of the raw data and transformed data of four quantitative traits. The subfigures in the diagonal show the histograms of the four quantitative traits (*R*-methadone, *S*-methadone, *R*-EDDP, and *S*-EDDP). The subfigures in the upper diagonal part are the pairwise scatterplots of the four quantitative traits. The subfigures in the lower diagonal part provide the pairwise Pearson correlation coefficients (in notation: r_P) and Spearman correlation coefficients (in notation: r_Sp) of the four quantitative traits. (**A**) The distribution of the raw data. (**B**) The distribution of the transformed data.
(TIF)

S6 Fig Genome-wide single-locus association test for plasma concentration of *S*-methadone, *R*-EDDP, and *S*-EDDP. Manhattan plot of genome-wide single-locus association test. The vertical axis indicates the raw *p* values of the association tests (in a scale of -log10). The horizontal axis is physical position of a SNP on different autosomes. Each point indicates the raw *p* value of a SNP. (**A**) Results of the analysis of plasma concentration of *S*-methadone. (**B**) Results of the analysis of plasma concentration of *R*-EDDP. (**C**) Results of the analysis of plasma concentration of *S*-EDDP.
(TIF)

S7 Fig Sliding-window genome-wide haplotype analysis of plasma concentration of *R*-EDDP and *S*-EDDP. Manhattan plot of genome-wide haplotype-based association test. The vertical axis is the raw *p* values of the omnibus haplotype tests (in a scale of -log10) and the horizontal axis is physical position of the initial SNP of a haplotype in different autosomes. Each point indicates the raw *p* value of a window. (**A**) Results of the analysis of plasma concentration of *R*-EDDP. (**B**) Results of the analysis of plasma concentration of *S*-EDDP.
(TIF)

S8 Fig Analysis flow of the replication study. (**A**) Flow of all statistical analyses. (**B**) Flow of quality control.
(TIF)

S9 Fig Gene structure and LD plot of 15 SNPs from the present genome-wide pharmacogenomic study and our previous candidate gene study of *CYP2B6*. In the top panel, coding regions on *CYP2B6* are displayed by green rectangles. In the bottom panel, pairwise LD of 15 SNPs on *CYP2B6* was measured by D’ [27]. SNPs with a strong LD were framed in a black inverse diamond block which was defined according to Gabriel’s confidence interval method [24].
(TIF)

S10 Fig Gene expression for *CYP2B6*, *SPON1*, and *GSG1L* in different tissues. The gene expression plots were generated from the Genotype-Tissue Expression (GTEx) Project [54] (http://www.gtexportal.org/home/). In each bar chart, the height of a bar indicates gene-level RPKM (i.e., reads per kilobase per million reads) values, from RNA sequencing experiments. (**A**) Gene expression of *CYP2B6*. (**B**) Gene expression of *SPON1*. (**C**) Gene expression of *GSG1L*.
(TIF)

S11 Fig Regional association plot and LD plot of the imputation region. Regional association plot and LD plot on the flanking region of rs17180299. In the regional association plot, the vertical axis is the raw *p* values of the association tests (in a scale of -log10) and the horizontal axis is physical position of SNPs (in unit of kb). Raw *p* values of single SNPs (orange circle) are shown. Rs17180299 is indicated by a square. In the LD plot, pairwise LD of SNPs was measured by D’ [27]. SNPs with a strong LD were framed in a black inverse diamond block which was defined according to Gabriel’s confidence interval method [24].
(TIF)

## References

[1] Mark TL, Woody GE, Juday T, Kleber HD. The economic costs of heroin addiction in the United States. Drug Alcohol Depend. 2001;61(2):195–206. Epub 2001/01/04. S0376-8716(00)00162-9 [pii]. . 11137285 10.1016/s0376-8716(00)00162-9

[2] Brown R. Heroin dependence. WMJ. 2004;103(4):20–6. Epub 2004/10/16. . 15481866

[3] Uchtenhagen A. Substitution management in opioid dependence. Journal of neural transmission Supplementum. 2003;(66):33–60. . 14582802 10.1007/978-3-7091-0541-2_3

[4] Mattick RP, Breen C, Kimber J, Davoli M. Methadone maintenance therapy versus no opioid replacement therapy for opioid dependence. The Cochrane database of systematic reviews. 2009;(3):CD002209 10.1002/14651858.CD002209.pub2 . 19588333 PMC7097731

[5] Mattick RP, Breen C, Kimber J, Davoli M. Methadone maintenance therapy versus no opioid replacement therapy for opioid dependence. The Cochrane database of systematic reviews. 2002;(4):CD002209 10.1002/14651858.CD002209 . 12519570

[6] Mattick RP, Breen C, Kimber J, Davoli M. Methadone maintenance therapy versus no opioid replacement therapy for opioid dependence. The Cochrane database of systematic reviews. 2003;(2):CD002209 10.1002/14651858.CD002209 . 12804430

[7] Gerber JG, Rhodes RJ, Gal J. Stereoselective metabolism of methadone N-demethylation by cytochrome P4502B6 and 2C19. Chirality. 2004;16(1):36–44. 10.1002/chir.10303 . 14628297

[8] Chang Y, Fang WB, Lin SN, Moody DE. Stereo-selective metabolism of methadone by human liver microsomes and cDNA-expressed cytochrome P450s: a reconciliation. Basic & clinical pharmacology & toxicology. 2011;108(1):55–62. 10.1111/j.1742-7843.2010.00628.x 20825389 PMC3005981

[9] Wang SC, Ho IK, Wu SL, Liu SC, Kuo HW, Lin KM, et al Development of a method to measure methadone enantiomers and its metabolites without enantiomer standard compounds for the plasma of methadone maintenance patients. Biomedical chromatography: BMC. 2010;24(7):782–8. 10.1002/bmc.1363 . 19904716

[10] Totah RA, Sheffels P, Roberts T, Whittington D, Thummel K, Kharasch ED. Role of CYP2B6 in stereoselective human methadone metabolism. Anesthesiology. 2008;108(3):363–74. 10.1097/ALN.0b013e3181642938 . 18292673

[11] Totah RA, Allen KE, Sheffels P, Whittington D, Kharasch ED. Enantiomeric metabolic interactions and stereoselective human methadone metabolism. The Journal of pharmacology and experimental therapeutics. 2007;321(1):389–99. 10.1124/jpet.106.117580 . 17259447

[12] Levran O, Peles E, Hamon S, Randesi M, Adelson M, Kreek MJ. CYP2B6 SNPs are associated with methadone dose required for effective treatment of opioid addiction. Addict Biol. 2013;18(4):709–16. 10.1111/j.1369-1600.2011.00349.x 21790905 PMC3735354

[13] Bunten H, Liang WJ, Pounder DJ, Seneviratne C, Osselton D. OPRM1 and CYP2B6 gene variants as risk factors in methadone-related deaths. Clinical pharmacology and therapeutics. 2010;88(3):383–9. 10.1038/clpt.2010.127 . 20668445

[14] Bunten H, Liang WJ, Pounder D, Seneviratne C, Osselton MD. CYP2B6 and OPRM1 gene variations predict methadone-related deaths. Addiction biology. 2011;16(1):142–4. . 21158011 10.1111/j.1369-1600.2010.00274.x

[15] Tsai HJ, Wang SC, Liu SW, Ho IK, Chang YS, Tsai YT, et al Assessment of CYP450 genetic variability effect on methadone dose and tolerance. Pharmacogenomics. 2014;15(7):977–86. Epub 2014/06/24. 10.2217/pgs.14.19 . 24956251

[16] Wang SC, Ho IK, Tsou HH, Tian JN, Hsiao CF, Chen CH, et al CYP2B6 polymorphisms influence the plasma concentration and clearance of the methadone S-enantiomer. Journal of clinical psychopharmacology. 2011;31(4):463–9. 10.1097/JCP.0b013e318222b5dd . 21694616

[17] Dobrinas M, Crettol S, Oneda B, Lahyani R, Rotger M, Choong E, et al Contribution of CYP2B6 alleles in explaining extreme (S)-methadone plasma levels: a CYP2B6 gene resequencing study. Pharmacogenetics and genomics. 2013;23(2):84–93. 10.1097/FPC.0b013e32835cb2e2 . 23249875

[18] Dennis BB, Bawor M, Thabane L, Sohani Z, Samaan Z. Impact of ABCB1 and CYP2B6 genetic polymorphisms on methadone metabolism, dose and treatment response in patients with opioid addiction: a systematic review and meta-analysis. PLoS ONE. 2014;9(1):e86114 Epub 2014/02/04. 10.1371/journal.pone.0086114 PONE-D-13-29310 [pii]. 24489693 PMC3906028

[19] Tsai HJ, Wang SC, Tian JN, Chang TK, Ho IK, Hsiao CF, et al PXR polymorphisms interacted with CYP2B6 polymorphisms on methadone metabolites. Journal of clinical psychopharmacology. 2013;33(1):137–40. 10.1097/01.jcp.0000426186.34421.de . 23288240

[20] Crettol S, Deglon JJ, Besson J, Croquette-Krokar M, Hammig R, Gothuey I, et al ABCB1 and cytochrome P450 genotypes and phenotypes: influence on methadone plasma levels and response to treatment. Clin Pharmacol Ther. 2006;80(6):668–81. Epub 2006/12/21. S0009-9236(06)00390-0 [pii] 10.1016/j.clpt.2006.09.012 . 17178267

[21] Fonseca F, de la Torre R, Diaz L, Pastor A, Cuyas E, Pizarro N, et al Contribution of cytochrome P450 and ABCB1 genetic variability on methadone pharmacokinetics, dose requirements, and response. PLoS ONE. 2011;6(5):e19527 Epub 2011/05/19. 10.1371/journal.pone.0019527 PONE-D-10-05589 [pii]. 21589866 PMC3093392

[22] Fredheim OM, Moksnes K, Borchgrevink PC, Kaasa S, Dale O. Clinical pharmacology of methadone for pain. Acta anaesthesiologica Scandinavica. 2008;52(7):879–89. Epub 2008/03/12. 10.1111/j.1399-6576.2008.01597.x AAS1597 [pii]. . 18331375

[23] Massey FJ. The Kolmogorov-Smirnov test for goodness of fit. J Amer Statist Ass. 1951;46(253):68–78. WOS:A1951UY90300007.

[24] Gabriel SB, Schaffner SF, Nguyen H, Moore JM, Roy J, Blumenstiel B, et al The structure of haplotype blocks in the human genome. Science. 2002;296(5576):2225–9. 10.1126/science.1069424 WOS:000176379000060. 12029063

[25] Wang Y, Fong PY, Leung FC, Mak W, Sham PC. Increased gene coverage and Alu frequency in large linkage disequilibrium blocks of the human genome. Gen Mol Res. 2007;6(4):1131–41. Epub 2008/02/15. gmr359 [pii]. . 18273807

[26] Benjamini Y, Hochberg Y. Controlling the false discovery rate: A practical and powerful approach to multiple testing. J R Statist Soc B. 1995;57(1):289–300. WOS:A1995QE45300017.

[27] Lewontin RC. The detection of linkage disequilibrium in molecular sequence data. Genetics. 1995;140(1):377–88. WOS:A1995QV52100031. 7635301 10.1093/genetics/140.1.377 PMC1206563

[28] Leger P, Dillingham R, Beauharnais CA, Kashuba AD, Rezk NL, Fitzgerald DW, et al CYP2B6 variants and plasma efavirenz concentrations during antiretroviral therapy in Port-au-Prince, Haiti. J Infect Dis. 2009;200(6):955–64. Epub 2009/08/08. 10.1086/605126 19659438 PMC2754599

[29] Sukasem C, Cressey TR, Prapaithong P, Tawon Y, Pasomsub E, Srichunrusami C, et al Pharmacogenetic markers of CYP2B6 associated with efavirenz plasma concentrations in HIV-1 infected Thai adults. Br J Clin Pharmacol. 2012;74(6):1005–12. Epub 2012/04/05. 10.1111/j.1365-2125.2012.04288.x 22471906 PMC3522814

[30] Salem AH, Fletcher CV, Brundage RC. Pharmacometric characterization of efavirenz developmental pharmacokinetics and pharmacogenetics in HIV-infected children. Antimicrobial agents and chemotherapy 2014;58(1):136–43. Epub 2013/10/23. 10.1128/AAC.01738-13 AAC.01738-13 [pii]. 24145522 PMC3910794

[31] Westerink WM, Schoonen WG. Cytochrome P450 enzyme levels in HepG2 cells and cryopreserved primary human hepatocytes and their induction in HepG2 cells. Toxicol In Vitro. 2007;21(8):1581–91. Epub 2007/07/20. S0887-2333(07)00171-3 [pii] 10.1016/j.tiv.2007.05.014 . 17637504

[32] Tolson AH, Li H, Eddington ND, Wang H. Methadone induces the expression of hepatic drug-metabolizing enzymes through the activation of pregnane X receptor and constitutive androstane receptor. Drug Metab Disposition. 2009;37(9):1887–94. Epub 2009/06/13. 10.1124/dmd.109.027854 dmd.109.027854 [pii]. 19520773 PMC2729327

[33] Sumioka A. Auxiliary subunits provide new insights into regulation of AMPA receptor trafficking. Journal of Biochemistry. 2013;153(4):331–7. Epub 2013/02/22. 10.1093/jb/mvt015 mvt015 [pii]. . 23426437

[34] Wang SC, Ho IK, Tsou HH, Liu SW, Hsiao CF, Chen CH, et al Functional genetic polymorphisms in CYP2C19 gene in relation to cardiac side effects and treatment dose in a methadone maintenance cohort. Omics: a journal of integrative biology. 2013;17(10):519–26. Epub 2013/09/11. 10.1089/omi.2012.0068 24016178 PMC3783925

[35] Kristensen K, Blemmer T, Angelo HR, Christrup LL, Drenck NE, Rasmussen SN, et al Stereoselective pharmacokinetics of methadone in chronic pain patients. Therapeutic drug monitoring. 1996;18(3):221–7. Epub 1996/06/01. . 8738759 10.1097/00007691-199606000-00001

[36] Ansermot N, Albayrak Ö, Schläpfer J, Crettol S, Croquette-Krokar M, Bourquin M, et al Substitution of (R, S)-methadone by (R)-methadone: impact on QTc interval. Archives of internal medicine. 2010;170(6):529–36. 10.1001/archinternmed.2010.26 20308640

[37] van Leeuwen EM, Kanterakis A, Deelen P, Kattenberg MV, Slagboom PE, de Bakker PI, et al Population-specific genotype imputations using minimac or IMPUTE2. Nat Protoc. 2015;10(9):1285–96. Epub 2015/08/01. 10.1038/nprot.2015.077 nprot.2015.077 [pii]. . 26226460

[38] Sun L, Dimitromanolakis A, Faye LL, Paterson AD, Waggott D, Bull SB. BR-squared: a practical solution to the winner's curse in genome-wide scans. Hum Genet. 2011;129(5):545–52. Epub 2011/01/20. 10.1007/s00439-011-0948-2 21246217 PMC3074069

[39] Horvath S. DNA methylation age of human tissues and cell types. Genome Biol. 2013;14(10):R115 Epub 2013/10/22. gb-2013-14-10-r115 [pii] 10.1186/gb-2013-14-10-r115 24138928 PMC4015143

[40] Howie BN, Donnelly P, Marchini J. A flexible and accurate genotype imputation method for the next generation of genome-wide association studies. PLoS Genet. 2009;5(6):e1000529 Epub 2009/06/23. 10.1371/journal.pgen.1000529 19543373 PMC2689936

[41] Gauderman WJ, Morrison JM. QUANTO 1.1: A computer program for power and sample size calculations for genetic-epidemiology studies. http://hydrauscedu/gxe. 2006.

[42] Chen CH, Wang SC, Tsou HH, Ho IK, Tian JN, Yu CJ, et al Genetic polymorphisms in CYP3A4 are associated with withdrawal symptoms and adverse reactions in methadone maintenance patients. Pharmacogenomics. 2011;12(10):1397–406. Epub 2011/09/10. 10.2217/pgs.11.103 . 21902501

[43] Wang SC, Ho IK, Tsou HH, Tian JN, Hsiao CF, Chen CH, et al CYP2B6 polymorphisms influence the plasma concentration and clearance of the methadone S-enantiomer. Journal of Clinical Psychopharmacology. 2011;31(4):463–9. Epub 2011/06/23. 10.1097/JCP.0b013e318222b5dd . 21694616

[44] Anderson CA, Pettersson FH, Clarke GM, Cardon LR, Morris AP, Zondervan KT. Data quality control in genetic case-control association studies. Nat Protoc. 2010;5(9):1564–73. Epub 2010/11/19. nprot.2010.116 [pii] 10.1038/nprot.2010.116 21085122 PMC3025522

[45] Wigginton JE, Cutler DJ, Abecasis GR. A note on exact tests of Hardy-Weinberg equilibrium. Am J Hum Genet. 2005;76(5):887–93. WOS:000228198300016. 15789306 10.1086/429864 PMC1199378

[46] Yang HC, Chang LC, Huggins RM, Chen CH, Mullighan CG. LOHAS: loss-of-heterozygosity analysis suite. Genet Epidemiol. 2011;35(4):247–60. Epub 2011/02/12. 10.1002/gepi.20573 . 21312262

[47] Purcell S, Neale B, Todd-Brown K, Thomas L, Ferreira MAR, Bender D, et al PLINK: A tool set for whole-genome association and population-based linkage analyses. Am J Hum Genet. 2007;81(3):559–75. 10.1086/519795 WOS:000249128200012. 17701901 PMC1950838

[48] Storey JD. A direct approach to false discovery rates. J Roy Stat Soc Ser B (Stat Method). 2002;64(3):479–98.

[49] Johnson AD, Handsaker RE, Pulit SL, Nizzari MM, O'Donnell CJ, de Bakker PI. SNAP: a web-based tool for identification and annotation of proxy SNPs using HapMap. Bioinformatics. 2008;24(24):2938–9. 10.1093/bioinformatics/btn564 18974171 PMC2720775

[50] Excoffier L, Slatkin M. Maximum-likelihood estimation of molecular haplotype frequencies in a diploid population. Mol Biol Evol. 1995;12(5):921–7. WOS:A1995RQ66400021. 7476138 10.1093/oxfordjournals.molbev.a040269

[51] Barrett JC, Fry B, Maller J, Daly MJ. Haploview: Analysis and visualization of LD and haplotype maps. Bioinformatics. 2005;21(2):263–5. 10.1093/bioinformatics/bth457 WOS:000226308500016. 15297300

[52] Fisher RA. Statistical Methods for Research Workers. London: Oliver and Boyd; 1925.

[53] Frazer KA, Ballinger DG, Cox DR, Hinds DA, Stuve LL, Gibbs RA, et al A second generation human haplotype map of over 3.1 million SNPs. Nature. 2007;449(7164):851–61. 10.1038/nature06258 WOS:000250230600036. 17943122 PMC2689609

[54] The GTEx Consortium. The Genotype-Tissue Expression (GTEx) project. Nat Genet. 2013;45(6):580–5. Epub 2013/05/30. 10.1038/ng.2653 ng.2653 [pii]. . 23715323 PMC4010069

