# Supplementary material for: Genome-Wide Pharmacogenomic Study on Methadone Maintenance Treatment Identifies SNP rs17180299 and Multiple Haplotypes on CYP2B6, SPON1, and GSG1L Associated with Plasma Concentrations of Methadone R- and S-enantiomers in Heroin-Dependent Patients
Source: PLoS Genet. 2016 Mar 24;12(3):e1005910. doi: 10.1371/journal.pgen.1005910 (PMC4806848; doi:10.1371/journal.pgen.1005910)
Supplement: S1 Table — In terminology, the window width indicates the physical distance between the first and last SNPs in a window, and the window size indicates the number of SNPs in a window. For each window size of 2, 3, 4, 5, and 10 SNPs, the frequency of windows with an average width of 0–1 kb, 1–10 kb, 10–20 kb, 20–50 kb, and >50 kb in our study are provided. In the last column, the frequency of linkage disequilibrium (LD) blocks with an average width of 0–1 kb, 1–10 kb, 10–20 kb, 20–50 kb, and >50 kb in the HapMap Asian population are provided [24, 25]. (DOCX) [file pgen.1005910.s001.docx]

**S1 Table. Distributions of windows with different sizes and distributions of LD blocks with different sizes.** In terminology, the window width indicates the physical distance between the first and last SNPs in a window, and the window size indicates the number of SNPs in a window. For each window size of 2, 3, 4, 5, and 10 SNPs, the frequency of windows with an average width of 0-1 kb, 1-10 kb, 10-20 kb, 20-50 kb, and >50 kb in our study are provided. In the last column, the frequency of linkage disequilibrium (LD) blocks with an average width of 0-1 kb, 1-10 kb, 10-20 kb, 20-50 kb, and >50 kb in the HapMap Asian population are provided [[24](#_ENREF_24),[25](#_ENREF_25)].

|  | Data from this study | | | | | ^a^ Data from HapMap II |
| --- | --- | --- | --- | --- | --- | --- |
| Window size (kb) | 2 | 3 | 4 | 5 | 10 | LD block size |
| 0-1 kb | 0.32 | 0.08 | 0.02 | 0.01 | 0.00 | N.A. |
| 1-10 kb | 0.57 | 0.65 | 0.55 | 0.42 | 0.07 | 0.46 |
| 10-20 kb | 0.08 | 0.17 | 0.25 | 0.30 | 0.24 | 0.207 |
| 20-50 kb | 0.03 | 0.08 | 0.15 | 0.22 | 0.46 | 0.223 |
| >50 kb | 0.00 | 0.01 | 0.03 | 0.05 | 0.23 | 0.119 |

^a^ The data of the block size of LD in the HapMap II Asian population were collected based on 45 Japanese in Tokyo (JPT) and 45 Han Chinese in Beijing (CHB) samples in the HapMap II Project.
