# Supplementary material for: Genome-Wide Pharmacogenomic Study on Methadone Maintenance Treatment Identifies SNP rs17180299 and Multiple Haplotypes on CYP2B6, SPON1, and GSG1L Associated with Plasma Concentrations of Methadone R- and S-enantiomers in Heroin-Dependent Patients
Source: PLoS Genet. 2016 Mar 24;12(3):e1005910. doi: 10.1371/journal.pgen.1005910 (PMC4806848; doi:10.1371/journal.pgen.1005910)
Supplement: S2 Table — In accordance with the inclusion steps of haplotypes in each analysis of plasma concentration of R-methadone and S-methadone, we list the chromosome and linkage disequilibrium (LD) block where the haplotypes are located. Slope estimate and its standard error (s.e.) and the corresponding 95% confidence interval are provided in the final two columns. (DOCX) [file pgen.1005910.s002.docx]

**S2 Table. Effect sizes of the significant haplotypes we identified.** In accordance with the inclusion steps of haplotypes in each analysis of plasma concentration of *R*-methadone and *S*-methadone, we list the chromosome and linkage disequilibrium (LD) block where the haplotypes are located. Slope estimate and its standard error (s.e.) and the corresponding 95% confidence interval are provided in the final two columns.

| Quantitative traits | Chrom. | LD block | Significant haplotypes | Slope estimate (s.e.) | 95% Confidence interval |
| --- | --- | --- | --- | --- | --- |
| Transformed plasma *R*-methadone/dose (ng/ml/mg) | 9 | 1 | *TTC* | 0.6064 (0.1260) | [0.3585, 0.8543] |
|  | 9 | 2 | *GC* | 0.5973 (0.1247) | [0.3521, 0.8425] |
|  | 9 | 3 | *CCA* | 0.7012 (0.1253) | [0.4547, 0.9477] |
|  | 9 | 4 | *CGGCG* | 0.7244 (0.1264) | [0.4757, 0.9731] |
| Transformed plasma *S*-methadone/dose (ng/ml/mg) | 11 | 2 | *TTA* | 0.5195 (0.1053) | [0.3124, 0.7266] |
|  | 11 | 3 | *TC* | 0.3430 (0.0839) | [0.1780, 0.5081] |
|  | 16 | 1 | *TCACT* | 0.2477 (0.0791) | [0.0922, 0.4032] |
|  | 16 | 1 | *TCGCT* | 0.3671 (0.1158) | [0.1392, 0.5949] |
|  | 16 | 1 | *TCGTT* | -0.2968 0.1015 | [-0.4965, -0.0971] |
|  | 16 | 2 | *CTGC* | 0.3947 (0.1319) | [0.1354, 0.6541] |
|  | 16 | 2 | *TTAC* | -0.2284 (0.0786) | [-0.3831, -0.0737] |
|  | 19 | 2 | *AGC* | -0.3331 (0.0851) | [-0.5005, -0.1656] |
|  | 19 | 3 | *GT* | -0.4196 (0.0875) | [-0.5917, -0.2475] |
|  | 19 | 4 | *CTTCCGCAT* | -0.4247 (0.0923) | [-0.6062, -0.2432] |
|  | 19 | 4 | *TCTACGCAC* | 0.4170 (0.0971) | [0.2260, 0.6081] |
|  | 19 | 5 | *TAATCG* | -0.4087 (0.0786) | [-0.5634, -0.2541] |
|  | 19 | 5 | *TCCTTT* | 0.5059 (0.1122) | [0.2851, 0.7266] |
|  | 19 | 6 | *CTAAG* | 0.3441 (0.1004) | [0.1467, 0.5415] |
|  | 19 | 6 | *CTGAT* | -0.4260 (0.0803) | [-0.5839, -0.2680] |
|  | 19 | 7 | *CGCG* | 0.3430 (0.0769) | [0.1918, 0.4943] |
|  | 19 | 7 | *GAAG* | -0.3851 (0.0790) | [-0.5405, -0.2296] |
