# Supplementary material for: Genome-Wide Pharmacogenomic Study on Methadone Maintenance Treatment Identifies SNP rs17180299 and Multiple Haplotypes on CYP2B6, SPON1, and GSG1L Associated with Plasma Concentrations of Methadone R- and S-enantiomers in Heroin-Dependent Patients
Source: PLoS Genet. 2016 Mar 24;12(3):e1005910. doi: 10.1371/journal.pgen.1005910 (PMC4806848; doi:10.1371/journal.pgen.1005910)
Supplement: S3 Table — We list the chromosome, window, linkage disequilibrium (LD) block, and gene where the haplotypes are located. Haplotype frequencies and raw and adjusted p values of the significant haplotypes are provided in the final two columns. (DOCX) [file pgen.1005910.s003.docx]

**S3 Table. All haplotypes in association tests of individual haplotypes for plasma concentration of *R*-methadone.** We list the chromosome, window, linkage disequilibrium (LD) block, and gene where the haplotypes are located. Haplotype frequencies and raw and adjusted p-values of the significant haplotypes are provided in the final two columns.

| Chrom. | Window ^a^ | LD block | Gene | Haplotype | Haplotype frequency | p-value  (adjusted p-value) |
| --- | --- | --- | --- | --- | --- | --- |
| 4 | w1- | 1 | *---* | *AA (rs12502737, and rs12502742)* | 0.445 | 6.94E-01  (1.00E+00) |
| 4 | w1- | 1 | *---* | *AC (rs12502737, and rs12502742)* | 0.022 | 3.49E-01  (1.00E+00) |
| 4 | w1- | 1 | *---* | *CC (rs12502737, and rs12502742)* | 0.533 | 8.96E-01  (1.00E+00) |
| 4 | w1 | 2 | *---* | *CC (rs6841005, and rs4697534)* | 0.293 | 4.73E-01  (1.00E+00) |
| 4 | w1 | 2 | *---* | *TT (rs6841005, and rs4697534)* | 0.176 | 2.81E-01  (1.00E+00) |
| 4 | w1 | 2 | *---* | *CT (rs6841005, and rs4697534)* | 0.531 | 1.41E-01  (8.46E-01) |
| 9 | w1- | 1 | *---* | *CCA (rs10115245, rs62572435, and rs1572144)* | 0.493 | 1.52E-01  (1.00E+00) |
| 9 | w1- | 1 | *---* | *TTC (rs10115245, rs62572435, and rs1572144)* | 0.095 | 2.26E-06  (4.07E-05) |
| 9 | w1- | 1 | *---* | *TCC (rs10115245, rs62572435, and rs1572144)* | 0.095 | 4.46E-01  (1.00E+00) |
| 9 | w1- | 1 | *---* | *CCC (rs10115245, rs62572435, and rs1572144)* | 0.318 | 3.66E-01  (1.00E+00) |
| 9 | w1 | 2 | *---* | *AC (rs1333935, and rs1333934)* | 0.331 | 7.59E-01  (1.00E+00) |
| 9 | w1 | 2 | *---* | *GC (rs1333935, and rs1333934)* | 0.097 | 2.49E-06  (4.48E-05) |
| 9 | w1 | 2 | *---* | *GA (rs1333935, and rs1333934)* | 0.571 | 1.19E-02  (2.14E-01) |
| 9 | w1 | 3 | *---* | *CTG (rs4085128, rs4877551, and rs4877552)* | 0.468 | 1.17E-01  (1.00E+00) |
| 9 | w1 | 3 | *---* | *TCA (rs4085128, rs4877551, and rs4877552)* | 0.437 | 1.08E-01  (1.00E+00) |
| 9 | w1 | 3 | *---* | *CCA (rs4085128, rs4877551, and rs4877552)* | 0.093 | 4.55E-08  (8.19E-07) |
| 9 | w2 ~ w3 | 4 | *---* | *TAGCA (rs970905, rs17180299, rs12000653, rs11138610, and rs72744905)* | 0.215 | 7.15E-01  (1.00E+00) |
| 9 | w2 ~ w3 | 4 | *---* | *CAGAG (rs970905, rs17180299, rs12000653, rs11138610, and rs72744905)* | 0.337 | 6.65E-01  (1.00E+00) |
| 9 | w2 ~ w3 | 4 | *---* | *TATCG (rs970905, rs17180299, rs12000653, rs11138610, and rs72744905)* | 0.226 | 2.52E-01  (1.00E+00) |
| 9 | w2 ~ w3 | 4 | *---* | *CGGCG (rs970905, rs17180299, rs12000653, rs11138610, and rs72744905)* | 0.090 | 2.24E-08  (4.03E-07) |
| 9 | w2 ~ w3 | 4 | *---* | *TAGCG (rs970905, rs17180299, rs12000653, rs11138610, and rs72744905)* | 0.125 | 5.43E-03  (9.77E-02) |
| 9 | w3+ | 5 | *---* | *GAC (rs7869484, rs7869210, and rs7020178)* | 0.410 | 5.35E-02  (9.63E-01) |
| 9 | w3+ | 5 | *---* | *AGT (rs7869484, rs7869210, and rs7020178)* | 0.097 | 1.10E-01  (1.00E+00) |
| 9 | w3+ | 5 | *---* | *GAT (rs7869484, rs7869210, and rs7020178)* | 0.492 | 3.61E-01  (1.00E+00) |

^a^ Notation “-” and “+” indicates the upstream and downstream of a window respectively when we expanded a significant window to encompass the flanking region on either side.
