# Supplementary material for: Genome-Wide Pharmacogenomic Study on Methadone Maintenance Treatment Identifies SNP rs17180299 and Multiple Haplotypes on CYP2B6, SPON1, and GSG1L Associated with Plasma Concentrations of Methadone R- and S-enantiomers in Heroin-Dependent Patients
Source: PLoS Genet. 2016 Mar 24;12(3):e1005910. doi: 10.1371/journal.pgen.1005910 (PMC4806848; doi:10.1371/journal.pgen.1005910)
Supplement: S4 Table — We list the chromosome, window, linkage disequilibrium (LD) block, and gene where the haplotypes are located. Haplotype frequencies and raw and adjusted p values of the significant haplotypes are provided in the final two columns. (DOCX) [file pgen.1005910.s004.docx]

**S4 Table. All haplotypes in association tests of individual haplotypes for plasma concentration of *S*-methadone.** We list the chromosome, window, linkage disequilibrium (LD) block, and gene where the haplotypes are located. Haplotype frequencies and raw and adjusted p-values of the significant haplotypes are provided in the final two columns.

| Chrom. | Window ^a^ | LD block | Gene | Haplotype | Haplotype frequency | p-value |
| --- | --- | --- | --- | --- | --- | --- |
| 11 | w1 | 1 | *SPON1* | *GCGGGT (rs10832203, rs10832223, rs10766163, rs11023133, AX-16534561, and rs10832231)* | 0.080 | 8.53E-01  (1.00E+00) |
| 11 | w1 | 1 | *SPON1* | *GTGGGT (rs10832203, rs10832223, rs10766163, rs11023133, AX-16534561, and rs10832231)* | 0.024 | 3.27E-01  (1.00E+00) |
| 11 | w1 | 1 | *SPON1* | *ATACAC (rs10832203, rs10832223, rs10766163, rs11023133, AX-16534561, and rs10832231)* | 0.110 | 3.50E-01  (1.00E+00) |
| 11 | w1 | 1 | *SPON1* | *GTGCGC (rs10832203, rs10832223, rs10766163, rs11023133, AX-16534561, and rs10832231)* | 0.317 | 1.27E-02  (1.52E-01) |
| 11 | w1 | 1 | *SPON1* | *ATACGC (rs10832203, rs10832223, rs10766163, rs11023133, AX-16534561, and rs10832231)* | 0.454 | 1.73E-02  (2.08E-01) |
| 11 | w1+ | 2 | *SPON1* | *GTG (rs4756776, rs4757240, and rs4756779)* | 0.128 | 9.24E-02  (1.00E+00) |
| 11 | w1+ | 2 | *SPON1* | *GTA (rs4756776, rs4757240, and rs4756779)* | 0.106 | 5.22E-01  (1.00E+00) |
| 11 | w1+ | 2 | *SPON1* | *TTA (rs4756776, rs4757240, and rs4756779)* | 0.153 | 1.27E-06  (1.52E-05) |
| 11 | w1+ | 2 | *SPON1* | *TGA (rs4756776, rs4757240, and rs4756779)* | 0.613 | 7.93E-03  (9.52E-02) |
| 11 | w1+ | 3 | *SPON1* | *TC (rs4757242, and rs7936301)* | 0.279 | 5.45E-05  (6.54E-04) |
| 11 | w1+ | 3 | *SPON1* | *AA (rs4757242, and rs7936301)* | 0.174 | 1.11E-01  (1.00E+00) |
| 11 | w1+ | 3 | *SPON1* | *TA (rs4757242, and rs7936301)* | 0.547 | 1.53E-02  (1.84E-01) |
| 16 | w1 ~ w2 | 1 | *GSG1L* | *TTGCC (rs60857987, rs713547, rs705912, rs4787995, and rs8182215)* | 0.196 | 2.14E-02  (1.71E-01) |
| 16 | w1 ~ w2 | 1 | *GSG1L* | *CCGTT (rs60857987, rs713547, rs705912, rs4787995, and rs8182215)* | 0.151 | 5.48E-01  (1.00E+00) |
| 16 | w1 ~ w2 | 1 | *GSG1L* | *TCGTT (rs60857987, rs713547, rs705912, rs4787995, and rs8182215)* | 0.154 | 3.54E-03  (2.83E-02) |
| 16 | w1 ~ w2 | 1 | *GSG1L* | *TCACT (rs60857987, rs713547, rs705912, rs4787995, and rs8182215)* | 0.368 | 1.73E-03  (1.38E-02) |
| 16 | w1 ~ w2 | 1 | *GSG1L* | *TCGCT (rs60857987, rs713547, rs705912, rs4787995, and rs8182215)* | 0.122 | 1.78E-03  (1.42E-02) |
| 16 | w2+ | 2 | *GSG1L* | *TTAC (AX-13044173, rs772972, rs772973, and rs11639671)* | 0.363 | 3.92E-03  (3.14E-02) |
| 16 | w2+ | 2 | *GSG1L* | *CTGC (AX-13044173, rs772972, rs772973, and rs11639671)* | 0.096 | 2.96E-03  (2.37E-02) |
| 16 | w2+ | 2 | *GSG1L* | *TCGT (AX-13044173, rs772972, rs772973, and rs11639671)* | 0.539 | 2.97E-01  (1.00E+00) |
| 19 | w1- | 1 | *CYP2A7P1* | *GGTGGA (rs72480748, AX-13442670, rs3852872, rs73034462, rs8108939, and rs4001941)* | 0.156 | 1.07E-01  (1.00E+00) |
| 19 | w1- | 1 | *CYP2A7P1* | *AATAGG (rs72480748, AX-13442670, rs3852872, rs73034462, rs8108939, and rs4001941)* | 0.054 | 9.81E-02  (1.00E+00) |
| 19 | w1- | 1 | *CYP2A7P1* | *AGTAGG (rs72480748, AX-13442670, rs3852872, rs73034462, rs8108939, and rs4001941)* | 0.238 | 5.00E-01  (1.00E+00) |
| 19 | w1- | 1 | *CYP2A7P1* | *GGTAGG (rs72480748, AX-13442670, rs3852872, rs73034462, rs8108939, and rs4001941)* | 0.026 | 1.31E-01  (1.00E+00) |
| 19 | w1- | 1 | *CYP2A7P1* | *GGCGCG (rs72480748, AX-13442670, rs3852872, rs73034462, rs8108939, and rs4001941)* | 0.307 | 3.21E-01  (1.00E+00) |
| 19 | w1- | 1 | *CYP2A7P1* | *GGTGCG (rs72480748, AX-13442670, rs3852872, rs73034462, rs8108939, and rs4001941)* | 0.219 | 2.83E-03  (1.27E-01) |
| 19 | w1- | 2 | *CYP2A7P1, CYP2B7P1* | *GGT (rs12461727, rs73038469, and rs4803406)* | 0.173 | 6.11E-02  (1.00E+00) |
| 19 | w1- | 2 | *CYP2A7P1, CYP2B7P1* | *GAC (rs12461727, rs73038469, and rs4803406)* | 0.226 | 3.81E-01  (1.00E+00) |
| 19 | w1- | 2 | *CYP2A7P1, CYP2B7P1* | *AGC (rs12461727, rs73038469, and rs4803406)* | 0.270 | 1.58E-04  (7.11E-03) |
| 19 | w1- | 2 | *CYP2A7P1, CYP2B7P1* | *GGC (rs12461727, rs73038469, and rs4803406)* | 0.328 | 2.86E-01  (1.00E+00) |
| 19 | w1 | 3 | *CYP2A7P1, CYP2B7P1* | *AC (rs8110485, and rs4124633)* | 0.323 | 1.80E-01  (1.00E+00) |
| 19 | w1 | 3 | *CYP2A7P1, CYP2B7P1* | *GT (rs8110485, and rs4124633)* | 0.259 | 2.43E-06  (1.09E-04) |
| 19 | w1 | 3 | *CYP2A7P1, CYP2B7P1* | *AT (rs8110485, and rs4124633)* | 0.419 | 5.38E-03  (2.42E-01) |
| 19 | w1 ~ w8 | 4 | *CYP2A7P1, CYP2B7P1* | *TCCCTGCGT (rs4560022, rs3889806, rs4803410, rs1017384, rs7251950, rs1808682, AX-13442755, rs3760657, and rs2054675)* | 0.122 | 3.00E-01  (1.00E+00) |
| 19 | w1 ~ w8 | 4 | *CYP2A7P1, CYP2B7P1* | *TCTCCGCAT (rs4560022, rs3889806, rs4803410, rs1017384, rs7251950, rs1808682, AX-13442755, rs3760657, and rs2054675)* | 0.052 | 7.60E-01  (1.00E+00) |
| 19 | w1 ~ w8 | 4 | *CYP2A7P1, CYP2B7P1* | *TCTCTACAT (rs4560022, rs3889806, rs4803410, rs1017384, rs7251950, rs1808682, AX-13442755, rs3760657, and rs2054675)* | 0.296 | 5.62E-01  (1.00E+00) |
| 19 | w1 ~ w8 | 4 | *CYP2A7P1, CYP2B7P1* | *CTTCCGCAT (rs4560022, rs3889806, rs4803410, rs1017384, rs7251950, rs1808682, AX-13442755, rs3760657, and rs2054675)* | 0.219 | 5.17E-06  (2.33E-04) |
| 19 | w1 ~ w8 | 4 | *CYP2A7P1, CYP2B7P1* | *CTTCCGAAT (rs4560022, rs3889806, rs4803410, rs1017384, rs7251950, rs1808682, AX-13442755, rs3760657, and rs2054675)* | 0.078 | 1.34E-01  (1.00E+00) |
| 19 | w1 ~ w8 | 4 | *CYP2A7P1, CYP2B7P1* | *TCTACGCAC (rs4560022, rs3889806, rs4803410, rs1017384, rs7251950, rs1808682, AX-13442755, rs3760657, and rs2054675)* | 0.179 | 2.31E-05  (1.04E-03) |
| 19 | w1 ~ w8 | 4 | *CYP2A7P1, CYP2B7P1* | *TCCCTGCAT (rs4560022, rs3889806, rs4803410, rs1017384, rs7251950, rs1808682, AX-13442755, rs3760657, and rs2054675)* | 0.019 | 4.52E-03  (2.03E-01) |
| 19 | w5 ~ w14 | 5 | *CYP2A7P1, CYP2B6* | *TCCTTT (rs8100458, rs7250601, rs7250991, rs11882424, rs8192719, and rs10853744)* | 0.126 | 9.04E-06  (4.07E-04) |
| 19 | w5 ~ w14 | 5 | *CYP2A7P1, CYP2B6* | *TACTTT (rs8100458, rs7250601, rs7250991, rs11882424, rs8192719, and rs10853744)* | 0.049 | 3.73E-03  (1.68E-01) |
| 19 | w5 ~ w14 | 5 | *CYP2A7P1, CYP2B6* | *TAACCG (rs8100458, rs7250601, rs7250991, rs11882424, rs8192719, and rs10853744)* | 0.056 | 9.42E-01  (1.00E+00) |
| 19 | w5 ~ w14 | 5 | *CYP2A7P1, CYP2B6* | *TCCTCG (rs8100458, rs7250601, rs7250991, rs11882424, rs8192719, and rs10853744)* | 0.016 | 7.17E-02  (1.00E+00) |
| 19 | w5 ~ w14 | 5 | *CYP2A7P1, CYP2B6* | *CAATCG (rs8100458, rs7250601, rs7250991, rs11882424, rs8192719, and rs10853744)* | 0.425 | 9.27E-02  (1.00E+00) |
| 19 | w5 ~ w14 | 5 | *CYP2A7P1, CYP2B6* | *TAATCG (rs8100458, rs7250601, rs7250991, rs11882424, rs8192719, and rs10853744)* | 0.311 | 2.03E-07  (9.14E-06) |
| 19 | w12 ~ w17 | 6 | *CYP2A7P1* | *TTAGG (rs61073883, rs1552222, rs7255904, AX-13442812, and rs11666982)* | 0.145 | 5.73E-02  (1.00E+00) |
| 19 | w12 ~ w17 | 6 | *CYP2A7P1* | *CTAAG (rs61073883, rs1552222, rs7255904, AX-13442812, and rs11666982)* | 0.179 | 6.81E-04  (3.06E-02) |
| 19 | w12 ~ w17 | 6 | *CYP2A7P1* | *CAGAT (rs61073883, rs1552222, rs7255904, AX-13442812, and rs11666982)* | 0.336 | 7.58E-01  (1.00E+00) |
| 19 | w12 ~ w17 | 6 | *CYP2A7P1* | *CTGAT (rs61073883, rs1552222, rs7255904, AX-13442812, and rs11666982)* | 0.330 | 2.04E-07  (9.18E-06) |
| 19 | w18 ~ w20 | 7 | *---* | *GGAA (rs17726861, rs7257703, rs17726963, and rs12982859)* | 0.209 | 5.44E-01  (1.00E+00) |
| 19 | w18 ~ w20 | 7 | *---* | *CGCG (rs17726861, rs7257703, rs17726963, and rs12982859)* | 0.392 | 1.11E-05  (5.00E-04) |
| 19 | w18 ~ w20 | 7 | *---* | *GAAG (rs17726861, rs7257703, rs17726963, and rs12982859)* | 0.337 | 2.08E-06  (9.36E-05) |
| 19 | w18 ~ w20 | 7 | *---* | *GGAG (rs17726861, rs7257703, rs17726963, and rs12982859)* | 0.062 | 1.56E-01  (1.00E+00) |
| 19 | w20 | 8 | *---* | *AG (rs58657125, and rs11672352)* | 0.247 | 4.34E-02  (1.00E+00) |
| 19 | w20 | 8 | *---* | *GG (rs58657125, and rs11672352)* | 0.030 | 2.25E-02  (1.00E+00) |
| 19 | w20 | 8 | *---* | *GA (rs58657125, and rs11672352)* | 0.717 | 1.92E-01  (1.00E+00) |
| 19 | w20+ | 9 | *CYP2G2P* | *TATTG (AX-13442856, rs1114979, rs8105353, rs7343168, and rs12461797)* | 0.216 | 6.72E-01  (1.00E+00) |
| 19 | w20+ | 9 | *CYP2G2P* | *TGGCG (AX-13442856, rs1114979, rs8105353, rs7343168, and rs12461797)* | 0.016 | 6.70E-01  (1.00E+00) |
| 19 | w20+ | 9 | *CYP2G2P* | *TGGTT (AX-13442856, rs1114979, rs8105353, rs7343168, and rs12461797)* | 0.023 | 5.08E-01  (1.00E+00) |
| 19 | w20+ | 9 | *CYP2G2P* | *GGGCT (AX-13442856, rs1114979, rs8105353, rs7343168, and rs12461797)* | 0.175 | 3.72E-01  (1.00E+00) |
| 19 | w20+ | 9 | *CYP2G2P* | *TGGCT (AX-13442856, rs1114979, rs8105353, rs7343168, and rs12461797)* | 0.547 | 1.91E-01  (1.00E+00) |
| 19 | w20+ | 10 | *---* | *TC (rs73563309, and rs177612)* | 0.066 | 8.40E-01  (1.00E+00) |
| 19 | w20+ | 10 | *---* | *CC (rs73563309, and rs177612)* | 0.262 | 5.55E-01  (1.00E+00) |
| 19 | w20+ | 10 | *---* | *CT (rs73563309, and rs177612)* | 0.672 | 6.59E-01  (1.00E+00) |

^a^ Notation “-” and “+” indicates the upstream and downstream of a window respectively when we expanded a significant window to encompass the flanking region on either side.
