# Supplementary material for: Genome-Wide Pharmacogenomic Study on Methadone Maintenance Treatment Identifies SNP rs17180299 and Multiple Haplotypes on CYP2B6, SPON1, and GSG1L Associated with Plasma Concentrations of Methadone R- and S-enantiomers in Heroin-Dependent Patients
Source: PLoS Genet. 2016 Mar 24;12(3):e1005910. doi: 10.1371/journal.pgen.1005910 (PMC4806848; doi:10.1371/journal.pgen.1005910)
Supplement: S5 Table — The number of individuals and means ± standard deviations (SD) of covariates and quantitative traits are provided by gender. In the final column, p values of the Kolmogorov-Smirnov Good-of-Fit tests for normality for the pre- and post-transformation data of four quantitative traits. (DOCX) [file pgen.1005910.s005.docx]

**S5 Table. Summary statistics of covariates and the raw data and transformed data of quantitative traits by gender.** The number of individuals (i.e., sample size), means ± standard deviations (SD) for covariates and quantitative traits are provided. The final column provides the p-value of the Kolmogorov-Smirnov Good-of-Fit test for normality for the raw data and transformed data of the two quantitative traits.

|  | **Male** | | **Female** | |  |
| --- | --- | --- | --- | --- | --- |
| **Characteristics** | **Sample size** | **Mean ± SD** | **Sample size** | **Mean ± SD** | **Normality test (p-value)** |
| Age (years) | 59 | 40.9292 ± 7.4602 | 17 | 35.1489 ± 5.6680 | - |
| BMI (kg/m^2^) | 59 | 23.8864 ± 3.9270 | 17 | 24.5353 ± 3.3292 | - |
| Raw plasma *R*-methadone/dose (ng/ml/mg) | 59 | 3.6028 ± 1.7612 | 17 | 3.8383 ± 2.2735 | 0.0102 |
| Raw plasma *S*-methadone/dose (ng/ml/mg) | 59 | 2.3956 ± 1.0153 | 17 | 2.4060 ± 1.7671 | 0.1903 |
| Transformed plasma *R*-methadone/dose (ng/ml/mg) | 59 | -0.0074 ± 0.9399 | 17 | 0.0257 ± 1.2182 | 0.4346 |
| Transformed plasma *S*-methadone/dose (ng/ml/mg) | 59 | 0.0457 ± 0.9564 | 17 | -0.1586 ± 1.1565 | 0.7813 |
