# Supplementary material for: Genome-Wide Pharmacogenomic Study on Methadone Maintenance Treatment Identifies SNP rs17180299 and Multiple Haplotypes on CYP2B6, SPON1, and GSG1L Associated with Plasma Concentrations of Methadone R- and S-enantiomers in Heroin-Dependent Patients
Source: PLoS Genet. 2016 Mar 24;12(3):e1005910. doi: 10.1371/journal.pgen.1005910 (PMC4806848; doi:10.1371/journal.pgen.1005910)
Supplement: S6 Table — We list the chromosome (Chrom.), linkage disequilibrium (LD) block, and significant haplotypes followed by their haplotype frequencies (HF) and raw p-values (P) at discovery stage and replication stage. The results at the replication stage are further stratified according to the urine morphine test (UMT): UMT = All, Negative, and Positive. (DOCX) [file pgen.1005910.s006.docx]

**S6 Table. Haplotype frequencies and *p*-values of the significant haplotypes, identified by our genome-wide pharmacogenomic study, at the discovery and replication stages.** We list the chromosome (Chrom.), linkage disequilibrium (LD) block, and significant haplotypes followed by their haplotype frequencies (HF) and raw *p*-values (P) at discovery stage and replication stage. The results at the replication stage are further stratified according to the urine morphine test (UMT): UMT = All, Negative, and Positive.

|  |  |  |  |  | Discovery stage | |  |  | Replication stage | | | |  |
| --- | --- | --- | --- | --- | --- | --- | --- | --- | --- | --- | --- | --- | --- |
|  |  |  |  |  | n = 344 | |  | UMT = All  n = 76 | | UMT = Negative  n = 44 | | UMT = Positive  n = 32 | |
| Transformed plasma concentration | Chrom. | LD block | Significant haplotype |  | HF | P |  | HF | P | HF | P | HF | P |
| *R*-methadone | 9 | 1 | *TTC* |  | 0.095 | 2.26E-06 |  | 0.059 | 0.358 | 0.068 | 0.587 | 0.047 | 0.590 |
| *R*-methadone | 9 | 2 | *GC* |  | 0.097 | 2.49E-06 |  | 0.092 | 0.283 | 0.091 | 0.476 | 0.094 | 0.454 |
| *R*-methadone | 9 | 3 | *CCA* |  | 0.093 | 4.55E-08 |  | 0.059 | 0.358 | 0.068 | 0.587 | 0.047 | 0.590 |
| *R*-methadone | 9 | 4 | *CGGCG* |  | 0.090 | 2.24E-08 |  | 0.059 | 0.358 | 0.068 | 0.587 | 0.047 | 0.590 |
| *S*-methadone | 11 | 2 | *TTA* |  | 0.153 | 1.27E-06 |  | 0.224 | 0.270 | 0.193 | 0.950 | 0.266 | **0.0294** |
| *S*-methadone | 11 | 3 | *TC* |  | 0.279 | 5.45E-05 |  | 0.303 | 0.379 | 0.273 | 0.848 | 0.344 | 0.172 |
| *S*-methadone | 16 | 1 | *TCACT* |  | 0.368 | 0.00173 |  | 0.362 | 0.989 | 0.364 | 0.874 | 0.359 | 0.769 |
| *S*-methadone | 16 | 1 | *TCGCT* |  | 0.122 | 0.00178 |  | 0.139 | 0.755 | 0.135 | 0.866 | 0.141 | 0.715 |
| *S*-methadone | 16 | 1 | *TCGTT* |  | 0.154 | 0.00354 |  | 0.125 | **0.044** | 0.172 | 0.0632 | 0.063 | 0.103 |
| *S*-methadone | 16 | 2 | *CTGC* |  | 0.096 | 0.00296 |  | 0.099 | 0.997 | 0.091 | 0.835 | 0.109 | 0.772 |
| *S*-methadone | 16 | 2 | *TTAC* |  | 0.363 | 0.00392 |  | 0.388 | 0.200 | 0.352 | 0.348 | 0.438 | 0.166 |
| *S*-methadone | 19 | 2 | *AGC* |  | 0.270 | 0.000158 |  | 0.229 | 0.876 | 0.225 | 0.767 | 0.234 | 0.705 |
| *S*-methadone | 19 | 3 | *GT* |  | 0.259 | 2.43E-06 |  | 0.217 | 0.84 | 0.250 | 0.322 | 0.172 | 0.575 |
| *S*-methadone | 19 | 4 | *CTTCCGCAT* |  | 0.219 | 5.17E-06 |  | 0.171 | **0.019** | 0.182 | **0.0397** | 0.172 | 0.370 |
| *S*-methadone | 19 | 4 | *TCTACGCAC* |  | 0.179 | 2.31E-05 |  | 0.204 | **0.026** | 0.205 | **0.0383** | 0.203 | 0.257 |
| *S*-methadone | 19 | 5 | *TAATCG* |  | 0.311 | 2.03E-07 |  | 0.276 | 0.519 | 0.284 | **0.0437** | 0.266 | 0.269 |
| *S*-methadone | 19 | 5 | *TCCTTT* |  | 0.126 | 9.04E-06 |  | 0.182 | 0.0877 | 0.177 | 0.290 | 0.187 | 0.135 |
| *S*-methadone | 19 | 6 | *CTAAG* |  | 0.179 | 0.000681 |  | 0.230 | 0.239 | 0.239 | 0.125 | 0.219 | 0.880 |
| *S*-methadone | 19 | 6 | *CTGAT* |  | 0.330 | 2.04E-07 |  | 0.349 | 0.286 | 0.364 | 0.0608 | 0.328 | 0.704 |
| *S*-methadone | 19 | 7 | *CGCG* |  | 0.392 | 1.11E-05 |  | 0.408 | 0.233 | 0.409 | 0.691 | 0.406 | 0.114 |
| *S*-methadone | 19 | 7 | *GAAG* |  | 0.337 | 2.08E-06 |  | 0.355 | 0.318 | 0.364 | 0.0608 | 0.344 | 0.624 |

*P*-values less than 0.05 at replication stage are shown in bold.
