# Supplementary material for: Genome-Wide Pharmacogenomic Study on Methadone Maintenance Treatment Identifies SNP rs17180299 and Multiple Haplotypes on CYP2B6, SPON1, and GSG1L Associated with Plasma Concentrations of Methadone R- and S-enantiomers in Heroin-Dependent Patients
Source: PLoS Genet. 2016 Mar 24;12(3):e1005910. doi: 10.1371/journal.pgen.1005910 (PMC4806848; doi:10.1371/journal.pgen.1005910)
Supplement: S7 Table — We list the chromosome (Chrom.), linkage disequilibrium (LD) block, and significant haplotypes followed by their p-values according to the urine morphine test (UMT): UMT = All, Negative, and Positive. (DOCX) [file pgen.1005910.s007.docx]

**S7 Table. Results of a meta-analysis by combining *p*-values at the discovery and replication stages.** We list the chromosome (Chrom.), linkage disequilibrium (LD) block, and significant haplotypes followed by their *p*-values according to the urine morphine test (UMT): UMT = All, Negative, and Positive.

| Transformed plasma concentration | Chrom. | LD block | Significant haplotype |  | *P*-value  UMT = All  (n = 344 + 76) | *P*-value  UMT = Negative  (n = 169 + 44) | *P*-value  UMT = Positive  (n = 173 + 32) |
| --- | --- | --- | --- | --- | --- | --- | --- |
| *R*-methadone | 9 | 1 | *TTC* |  | **1.22E-05** | **0.0069** | **0.0046** |
| *R*-methadone | 9 | 2 | *GC* |  | **1.07E-05** | **0.0057** | **0.0036** |
| *R*-methadone | 9 | 3 | *CCA* |  | **3.08E-07** | **0.0084** | **5.87E-05** |
| *R*-methadone | 9 | 4 | *CGGCG* |  | **1.58E-07** | **0.0056** | **4.83E-05** |
| *S*-methadone | 11 | 2 | *TTA* |  | **5.45E-06** | **0.0107** | **0.0001** |
| *S*-methadone | 11 | 3 | *TC* |  | **0.0002** | 0.0837 | **0.0028** |
| *S*-methadone | 16 | 1 | *TCACT* |  | **0.0126** | 0.5522 | **0.0106** |
| *S*-methadone | 16 | 1 | *TCGCT* |  | **0.0102** | **0.0105** | 0.5969 |
| *S*-methadone | 16 | 1 | *TCGTT* |  | **0.0015** | **0.0415** | **0.0083** |
| *S*-methadone | 16 | 2 | *CTGC* |  | **0.0201** | **0.0394** | 0.4193 |
| *S*-methadone | 16 | 2 | *TTAC* |  | **0.0064** | 0.0911 | 0.0705 |
| *S*-methadone | 19 | 2 | *AGC* |  | **0.0014** | **0.0408** | **0.0412** |
| *S*-methadone | 19 | 3 | *GT* |  | **2.88E-05** | **0.0037** | **0.0036** |
| *S*-methadone | 19 | 4 | *CTTCCGCAT* |  | **1.64E-06** | **4.35E-05** | **0.0178** |
| *S*-methadone | 19 | 4 | *TCTACGCAC* |  | **9.34E-06** | **0.0006** | **0.0475** |
| *S*-methadone | 19 | 5 | *TAATCG* |  | **1.80E-06** | **0.0003** | **0.0002** |
| *S*-methadone | 19 | 5 | *TCCTTT* |  | **1.19E-05** | **0.0050** | **0.0132** |
| *S*-methadone | 19 | 6 | *CTAAG* |  | **0.0016** | **0.0184** | 0.1169 |
| *S*-methadone | 19 | 6 | *CTGAT* |  | **1.03E-06** | **0.0003** | **0.0033** |
| *S*-methadone | 19 | 7 | *CGCG* |  | **3.59E-05** | **0.0109** | **0.0032** |
| *S*-methadone | 19 | 7 | *GAAG* |  | **1.01E-05** | **0.0009** | **0.0066** |

*P*-values less than 0.05 are shown in bold.
