# Supplementary figures and images for: Genome-Wide Pharmacogenomic Study on Methadone Maintenance Treatment Identifies SNP rs17180299 and Multiple Haplotypes on CYP2B6, SPON1, and GSG1L Associated with Plasma Concentrations of Methadone R- and S-enantiomers in Heroin-Dependent Patients
Source: PLoS Genet. 2016 Mar 24;12(3):e1005910. doi: 10.1371/journal.pgen.1005910 (PMC4806848; doi:10.1371/journal.pgen.1005910)

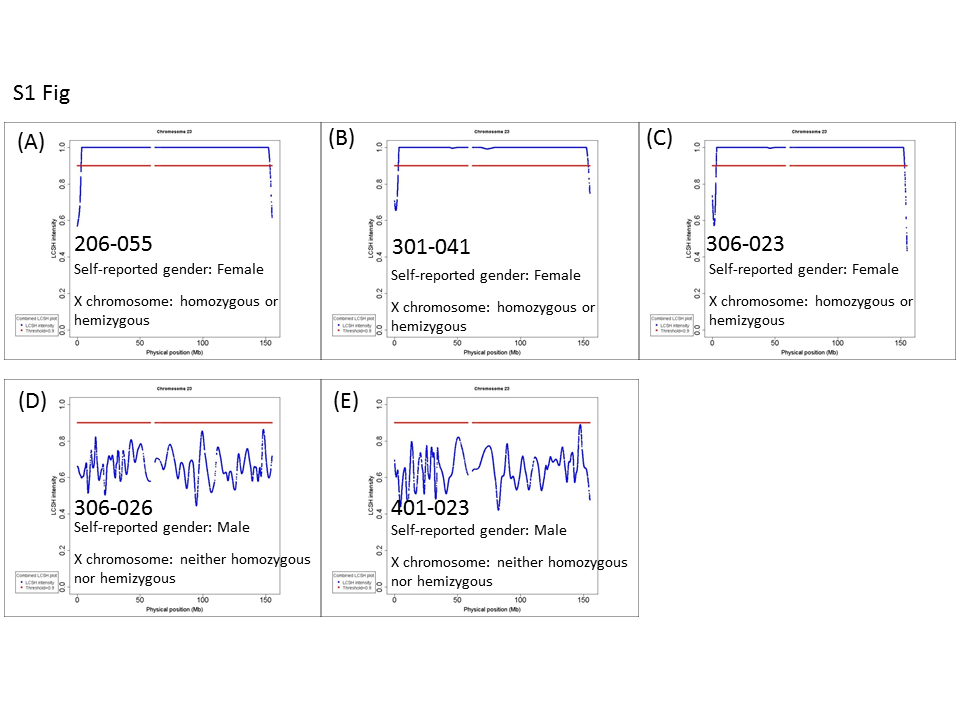

Supplement: S1 Fig — The homozygosity intensities of each individual are calculated and plotted based on 12,531 SNPs on chromosome X using software LOHAS. Five individuals exhibited a homozygosity pattern of X chromosome(s) inconsistent to their self-reported gender: (A) Individual 206–055: the self-report gender is “female” but the pattern of homozygosity intensity suggests that chromosome X is hemizygous or homozygous. (B) Individual 301–041: the self-report gender is “female” but the pattern of homozygosity intensity suggests that chromosome X is hemizygous or homozygous. (C) Individual 306–023: the self-report gender is “female” but the pattern of homozygosity intensity suggests that chromosome X is hemizygous or homozygous. (D) Individual 306–026: the self-report gender is “male” but the pattern of homozygosity intensity suggests that chromosome X is neither hemizygous nor homozygous. (E) Individual 401–023: the self-report gender is “male” but the pattern of homozygosity intensity suggests that chromosome X is neither hemizygous nor homozygous. (TIF) [file pgen.1005910.s008.tif]

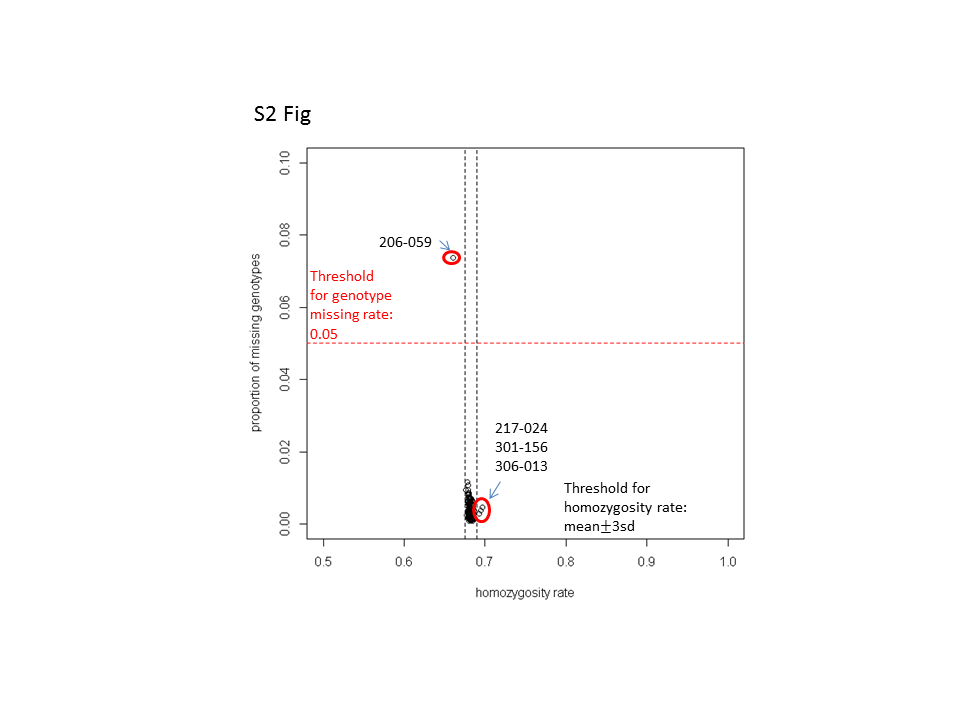

Supplement: S2 Fig — The genome-wide homozygosity rate (i.e., one minus heterozygosity rate) and proportion of missing genotype (i.e., one minus genotyping call rate) of each individual are calculated and plotted based on autosomal SNPs. The two vertical reference lines are the mean homozygosity rate ± 3 standard deviations. Four individuals are located outside the lower and upper reference lines of homozygosity rate, including three samples (217–024, 301–156, and 306–013) with an over-high genome-wide homozygosity rate and one sample (206–059) with an over-low genome-wide homozygosity rate are found. The sample (206–059) was already removed because of a low genotyping call rate of <0.95. (TIF) [file pgen.1005910.s009.tif]

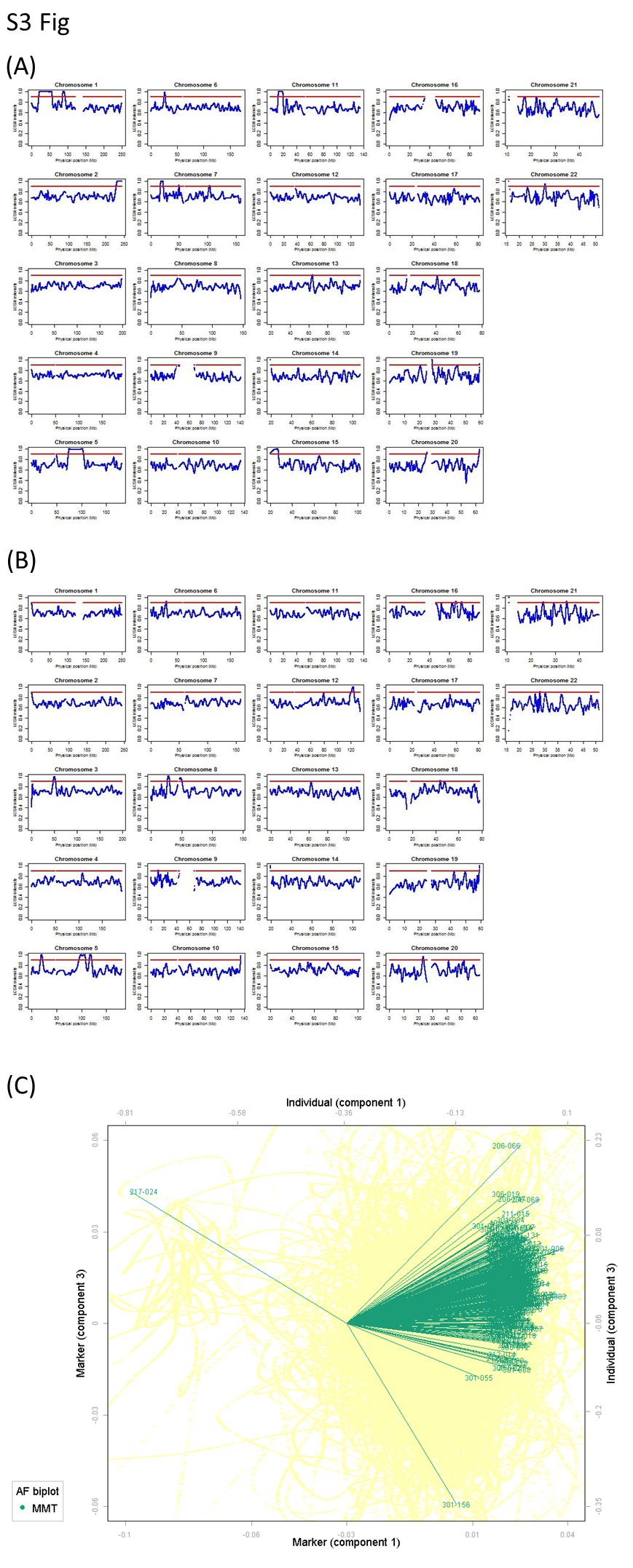

Supplement: S3 Fig — (A) Principal component plot of homozygosity intensity identified two outlier MMT patients (217–024 and 301–156) who carried multiple excessive runs of homozygosity on multiple chromosomes. (B) For patient 217–024, chromosome-wise homozygosity intensity plot found excessive runs of homozygosity on chromosome 1, 2, 5, 7, 11 and 15. (C) For patient 301–156, chromosome-wise homozygosity intensity plot found excessive runs of homozygosity on chromosome 5. Each of (B) and (C) consists of 22 subfigures. Each subfigure presents a homozygosity intensity plot for one autosomal chromosome. The vertical axis is the estimated homozygosity intensity, and the horizontal axis is physical position (Mb). Each point denotes an anchor SNP of a sliding window, and the gap in each subplot represents the centromeric gap. The reference line is a homozygosity intensity threshold of 0.9. (TIF) [file pgen.1005910.s010.tif]

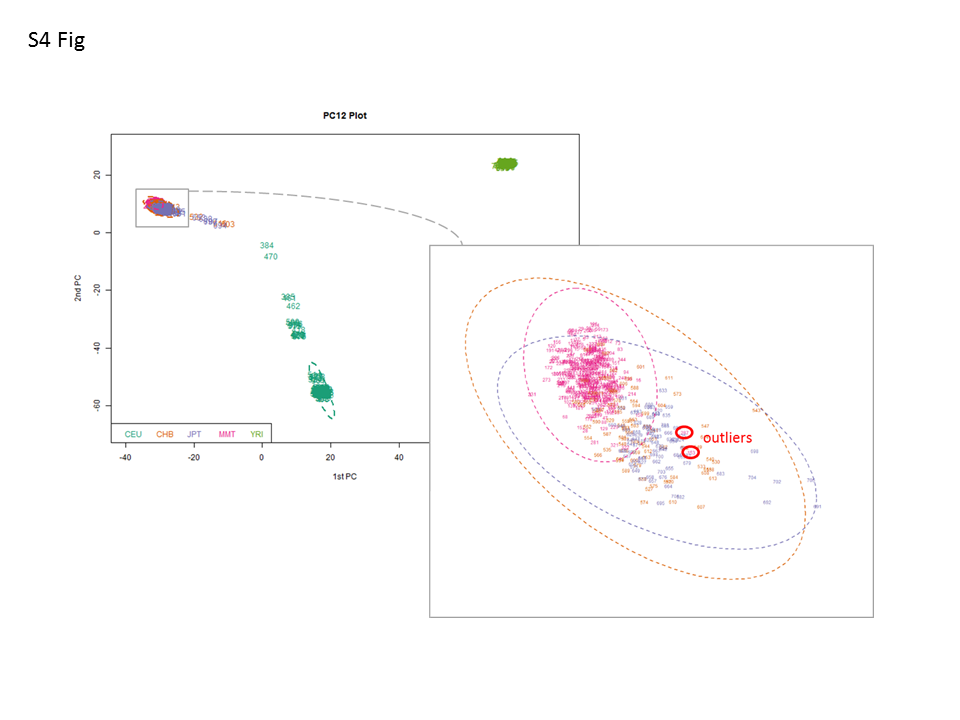

Supplement: S4 Fig — Principal component plot of 344 MMT patients in Taiwan (MMT; pink color) and 210 independent individuals from the International HapMap II Project [53]. The HapMap II individuals consisted of 30 married African couples from Yoruba in Ibadan (YRI; green color), 30 married Caucasian couples of European-descent residing in Utah (CEU; cyan color), and 90 Asian people, including 45 Han Chinese people in Beijing (CHB; orange color) and 45 Japanese people in Tokyo (JPT; purple color). The ellipse curves indicate the 99.99% confidence bands of the first two principal components of allele frequency. The individuals from African, European and Asian populations are well classified in the left-hand figure. The right-hand figure zooms in the cluster pattern of three East-Asian populations. In comparison, the MMT patients from Taiwan are closer to the CHB people than the JPT people. Two MMT patients (sample ID: 301–011 and 301–107) lie outside the confidence band and identified as divergent ancestry outliers of the MMT patients in Taiwan. (TIF) [file pgen.1005910.s011.tif]

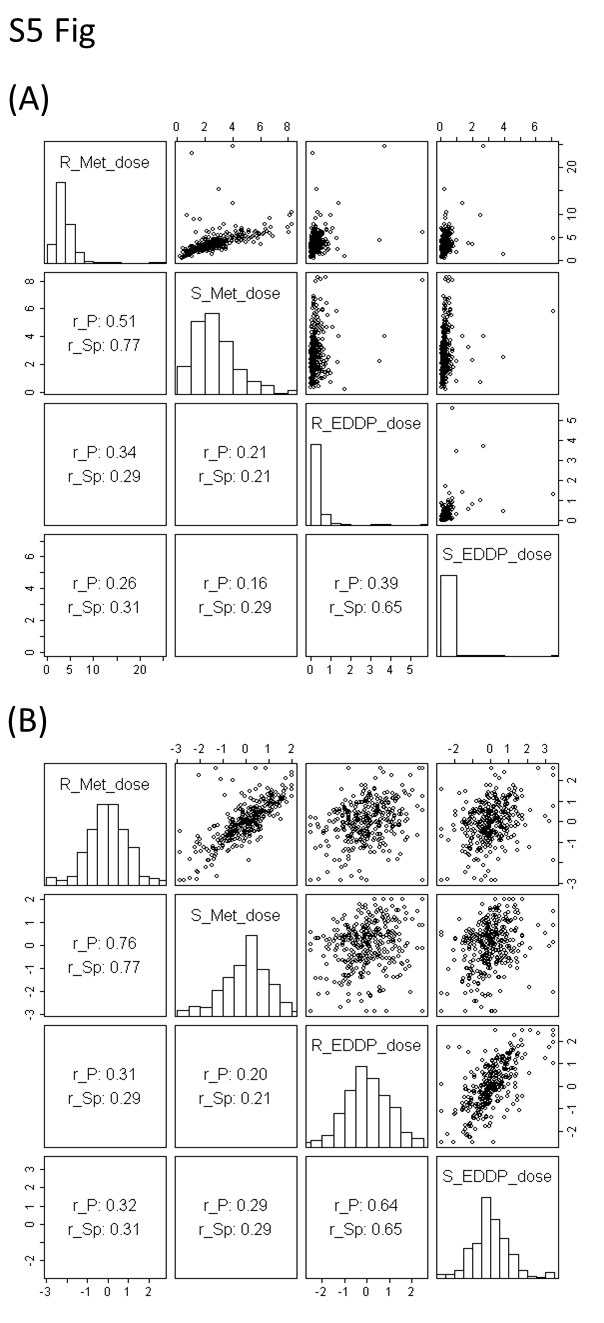

Supplement: S5 Fig — The subfigures in the diagonal show the histograms of the four quantitative traits (R-methadone, S-methadone, R-EDDP, and S-EDDP). The subfigures in the upper diagonal part are the pairwise scatterplots of the four quantitative traits. The subfigures in the lower diagonal part provide the pairwise Pearson correlation coefficients (in notation: r_P) and Spearman correlation coefficients (in notation: r_Sp) of the four quantitative traits. (A) The distribution of the raw data. (B) The distribution of the transformed data. (TIF) [file pgen.1005910.s012.tif]

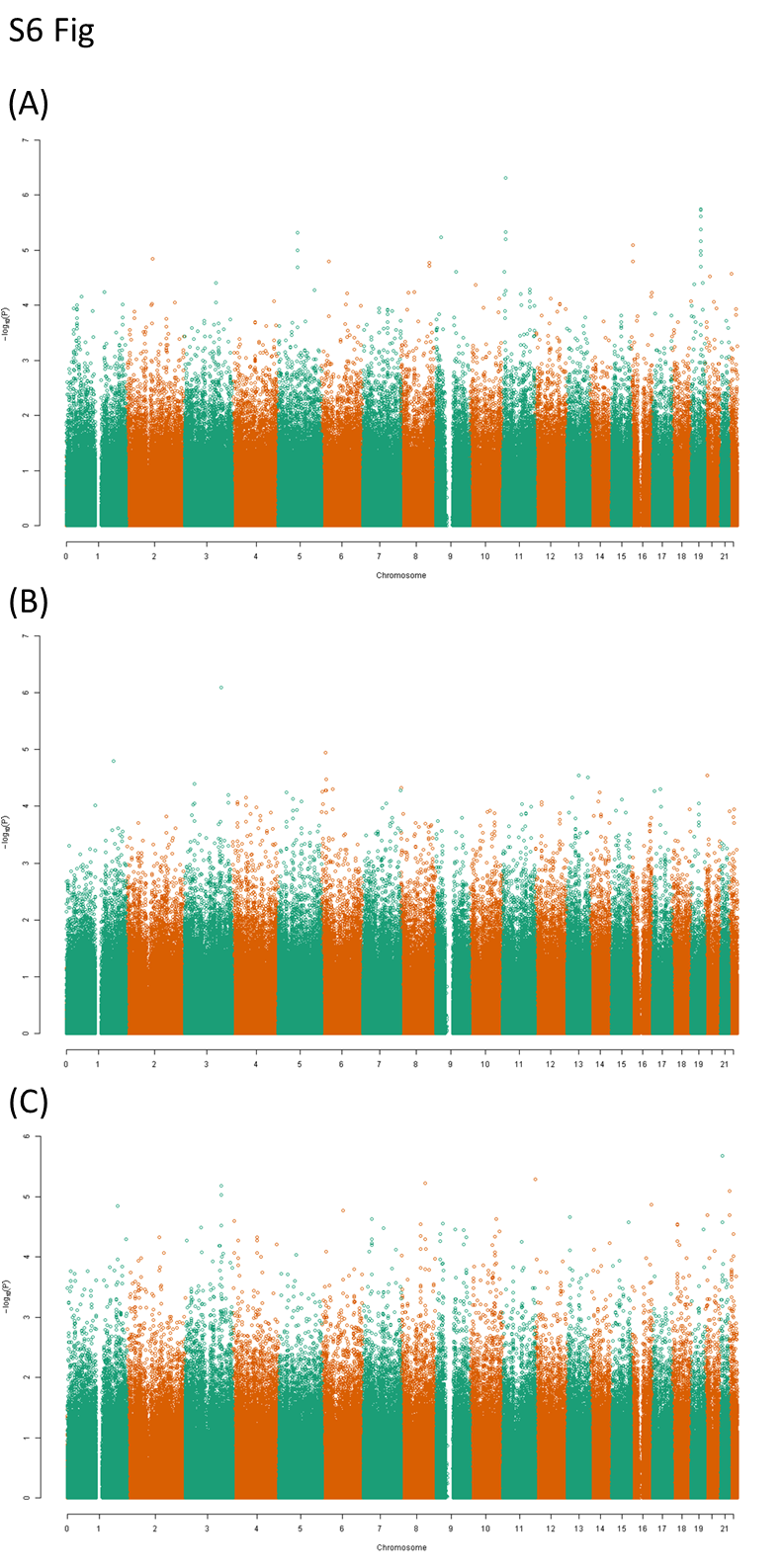

Supplement: S6 Fig — Manhattan plot of genome-wide single-locus association test. The vertical axis indicates the raw p values of the association tests (in a scale of -log10). The horizontal axis is physical position of a SNP on different autosomes. Each point indicates the raw p value of a SNP. (A) Results of the analysis of plasma concentration of S-methadone. (B) Results of the analysis of plasma concentration of R-EDDP. (C) Results of the analysis of plasma concentration of S-EDDP. (TIF) [file pgen.1005910.s013.tif]

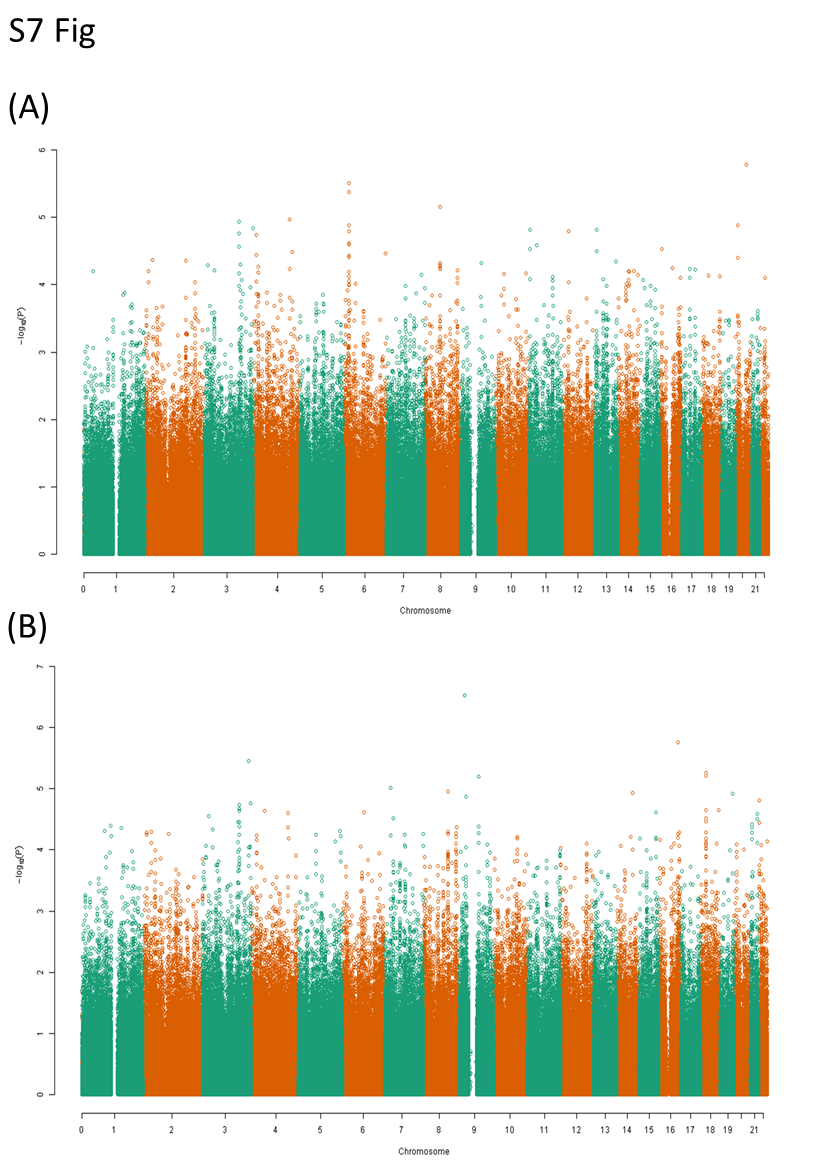

Supplement: S7 Fig — Manhattan plot of genome-wide haplotype-based association test. The vertical axis is the raw p values of the omnibus haplotype tests (in a scale of -log10) and the horizontal axis is physical position of the initial SNP of a haplotype in different autosomes. Each point indicates the raw p value of a window. (A) Results of the analysis of plasma concentration of R-EDDP. (B) Results of the analysis of plasma concentration of S-EDDP. (TIF) [file pgen.1005910.s014.tif]

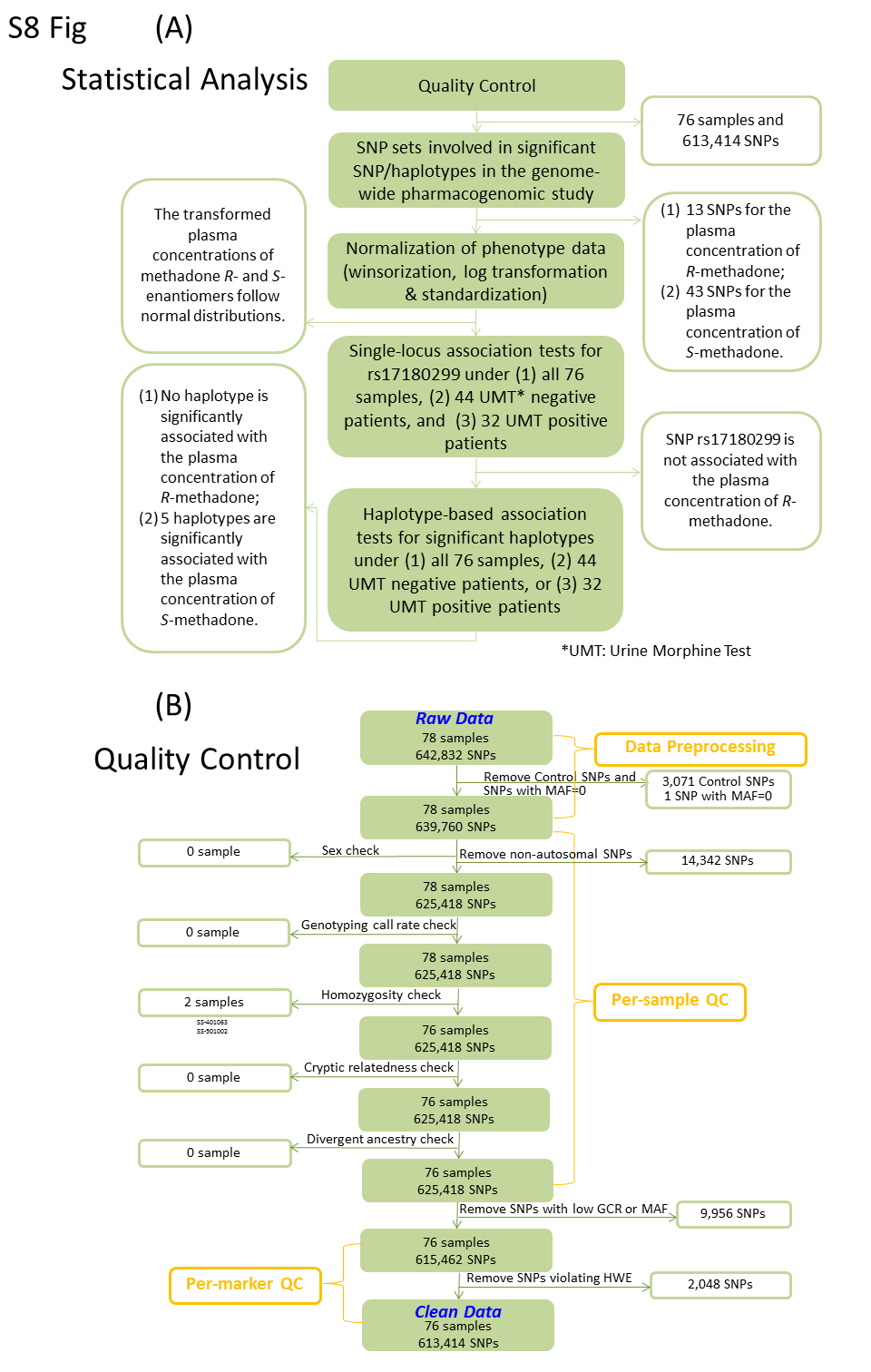

Supplement: S8 Fig — (A) Flow of all statistical analyses. (B) Flow of quality control. (TIF) [file pgen.1005910.s015.tif]

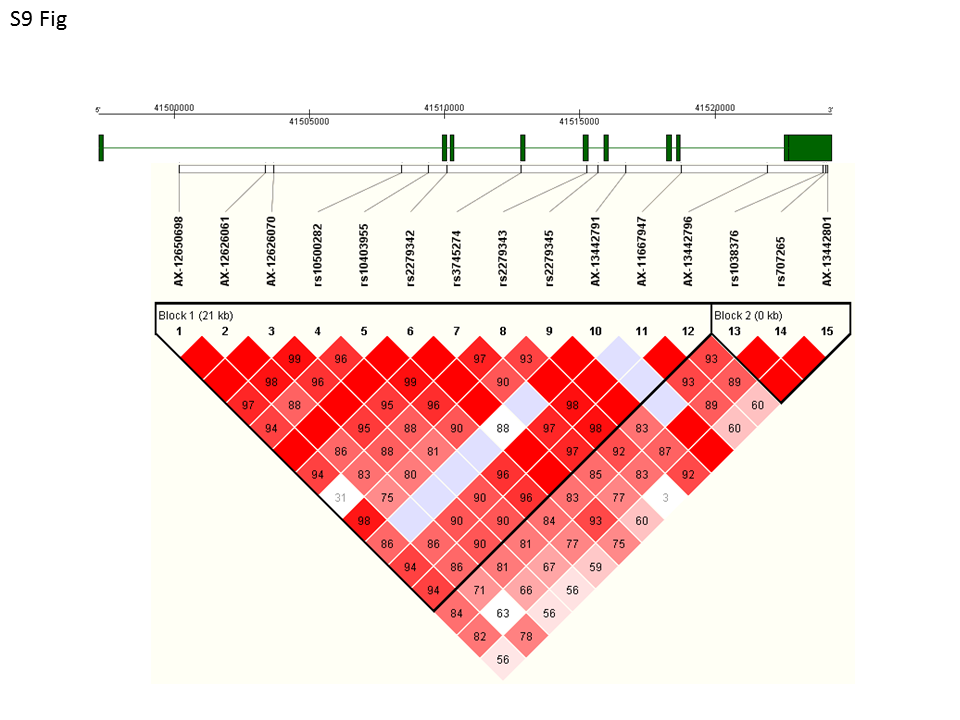

Supplement: S9 Fig — In the top panel, coding regions on CYP2B6 are displayed by green rectangles. In the bottom panel, pairwise LD of 15 SNPs on CYP2B6 was measured by D’ [27]. SNPs with a strong LD were framed in a black inverse diamond block which was defined according to Gabriel’s confidence interval method [24]. (TIF) [file pgen.1005910.s016.tif]

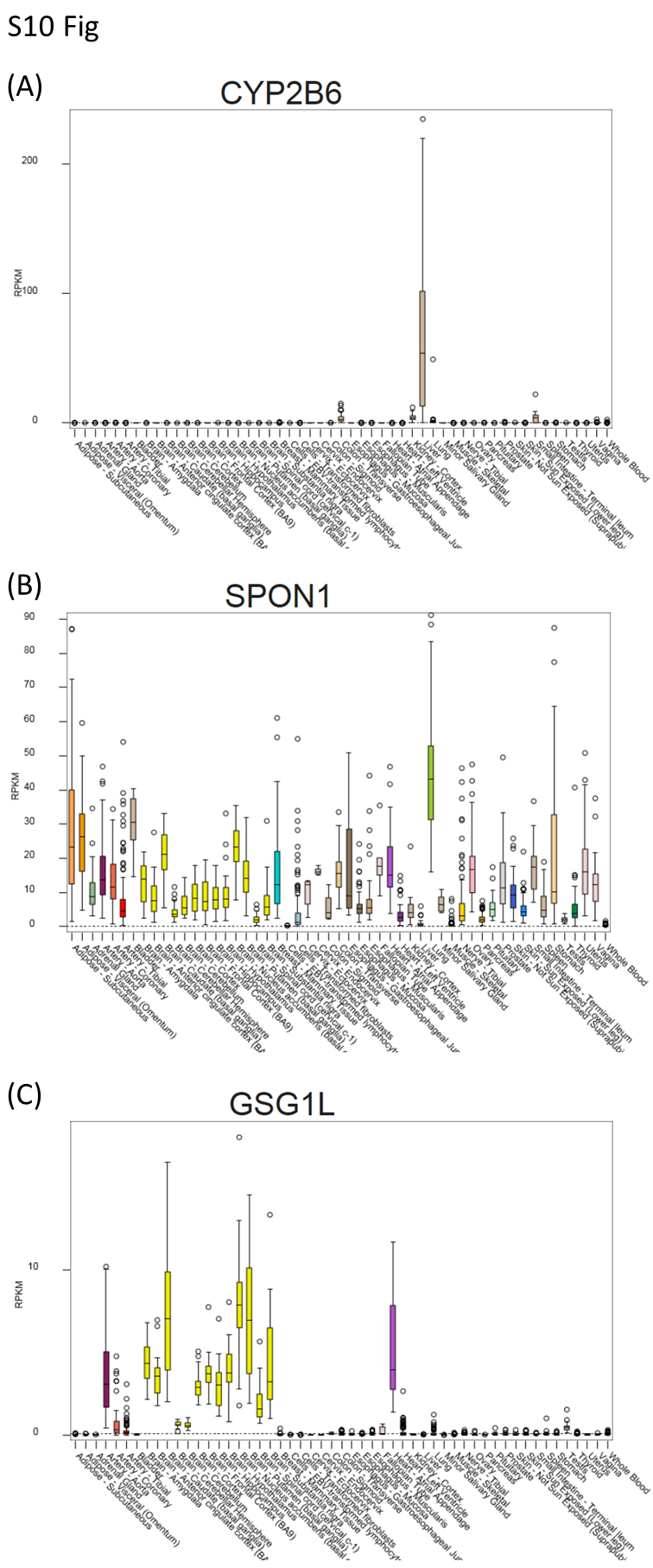

Supplement: S10 Fig — The gene expression plots were generated from the Genotype-Tissue Expression (GTEx) Project [54] (http://www.gtexportal.org/home/). In each bar chart, the height of a bar indicates gene-level RPKM (i.e., reads per kilobase per million reads) values, from RNA sequencing experiments. (A) Gene expression of CYP2B6. (B) Gene expression of SPON1. (C) Gene expression of GSG1L. (TIF) [file pgen.1005910.s017.tif]

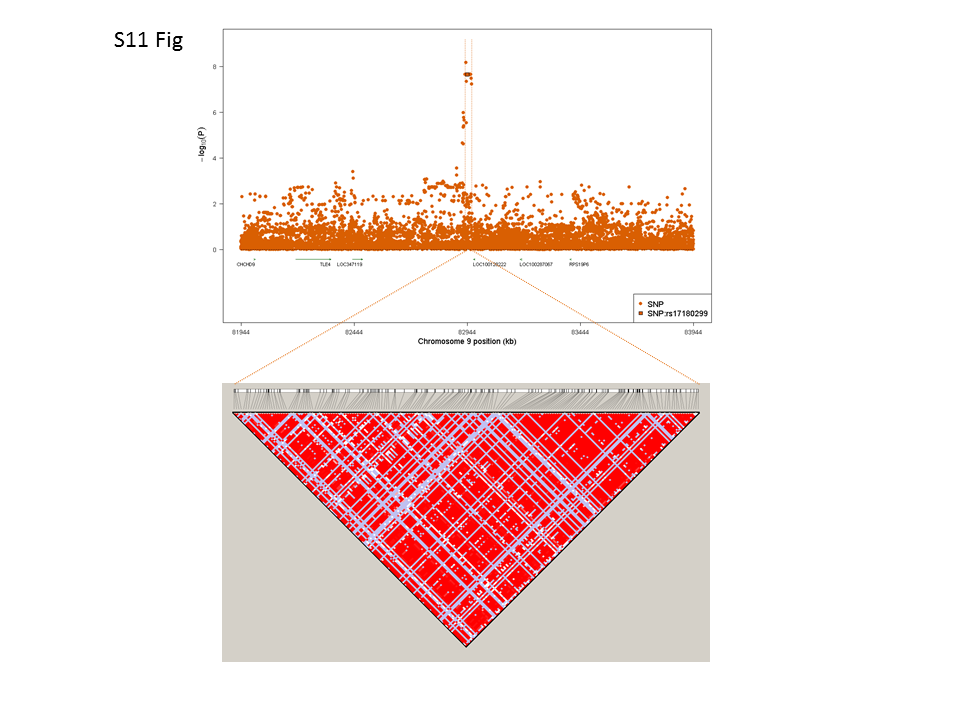

Supplement: S11 Fig — Regional association plot and LD plot on the flanking region of rs17180299. In the regional association plot, the vertical axis is the raw p values of the association tests (in a scale of -log10) and the horizontal axis is physical position of SNPs (in unit of kb). Raw p values of single SNPs (orange circle) are shown. Rs17180299 is indicated by a square. In the LD plot, pairwise LD of SNPs was measured by D’ [27]. SNPs with a strong LD were framed in a black inverse diamond block which was defined according to Gabriel’s confidence interval method [24]. (TIF) [file pgen.1005910.s018.tif]
